# Description of the Pliocene marsupial *Ambulator keanei* gen. nov. (Marsupialia: Diprotodontidae) from inland Australia and its locomotory adaptations

**DOI:** 10.1098/rsos.230211

**Published:** 2023-05-31

**Authors:** Jacob D. van Zoelen, Aaron B. Camens, Trevor H. Worthy, Gavin J. Prideaux

**Affiliations:** College of Science and Engineering, Flinders University, Bedford Park, SA 5042, Australia

**Keywords:** fossil marsupials, Diprotodontidae, Australia, Pliocene, megafauna, cranial and postcranial morphology

## Abstract

Diprotodontids were the largest marsupials to exist and an integral part of Australian terrestrial ecosystems until the last members of the group became extinct approximately 40 000 years ago. Despite the frequency with which diprotodontid remains are encountered, key aspects of their morphology, systematics, ecology and evolutionary history remain poorly understood. Here we describe new skeletal remains of the Pliocene taxon *Zygomaturus keanei* from northern South Australia. This is only the third partial skeleton of a late Cenozoic diprotodontid described in the last century, and the first displaying soft tissue structures associated with footpad impressions. Whereas it is difficult to distinguish *Z. keanei* and the type species of the genus, *Z. trilobus*, on dental grounds, the marked cranial and postcranial differences suggest that *Z. keanei* warrants genus-level distinction. Accordingly, we place it in the monotypic *Ambulator* gen. nov. We, also recognize the late Miocene *Z. gilli* as a *nomen dubium*. Features of the forelimb, manus and pes reveal that *Ambulator keanei* was more graviportal with greater adaptation to quadrupedal walking than earlier diprotodontids. These adaptations may have been driven by a need to travel longer distances to obtain resources as open habitats expanded in the late Pliocene of inland Australia.

## 1. Introduction

The extinct marsupial family Diprotodontidae consisted of large quadrupedal herbivores, with the largest species, *Diprotodon optatum* Owen, 1838, weighing in excess of two tonnes [1]. The family inhabited Australia and New Guinea for greater than 26 million years (Ma) until its complete extinction 50–40 ka (thousand years) ago [2–5]. Most systematic studies of diprotodontids have focused on the dentition [6–14], with few describing and comparing cranial or postcranial features [15]. Diprotodontid postcranial elements are relatively common in late Cenozoic fossil deposits, but have been described to date only for *D. optatum*, *Zygomaturus trilobus* Macleay, 1859 and *Hulitherium tomasettii* Flannery & Rich, 1986 from the Pleistocene [15–18], and *Eowenia grata* (DeVis, 1887) from the Pliocene Tirari Formation of northern South Australia [15–18]. This paper describes a partial skeleton of a second diprotodontid *Z. keanei* Stirton, 1967 [10] from the Tirari Formation.

The Tirari Formation is located in the Tirari Sub-basin of the Lake Eyre Basin [19] and represents one of the most geographically and temporally expansive vertebrate-bearing sequences of late Cenozoic age on the continent. Forming the primary basis for defining the Tirarian land mammal age, magnetic polarity stratigraphy and stage-of-evolution biochronology suggest an age range between 3.6 and 3.9 Ma [20,21]. The Tirari Formation is composed of three divisions: the basal (Mampuwordu Member) and overlying (Pompapillina Member) channel fills, and the intervening Main Body [20]. Vertebrate fossils have been described primarily from the channel fills to date, but do occur rarely within the Main Body. All four mainland Pliocene diprotodontid taxa are known from this formation: *Meniscolophus mawsoni* Stirton, 1955 [22], *Z. keanei* [10], *Euowenia grata* (De Vis, 1887) [23] and *Euryzygoma dunense* (De Vis, 1888) [24]. Diprotodontid postcranial material has been collected from many Tirari Formation sites, but only those of *Euo. grata* have hitherto been ascribed to a species [16].

**Figure 1.**
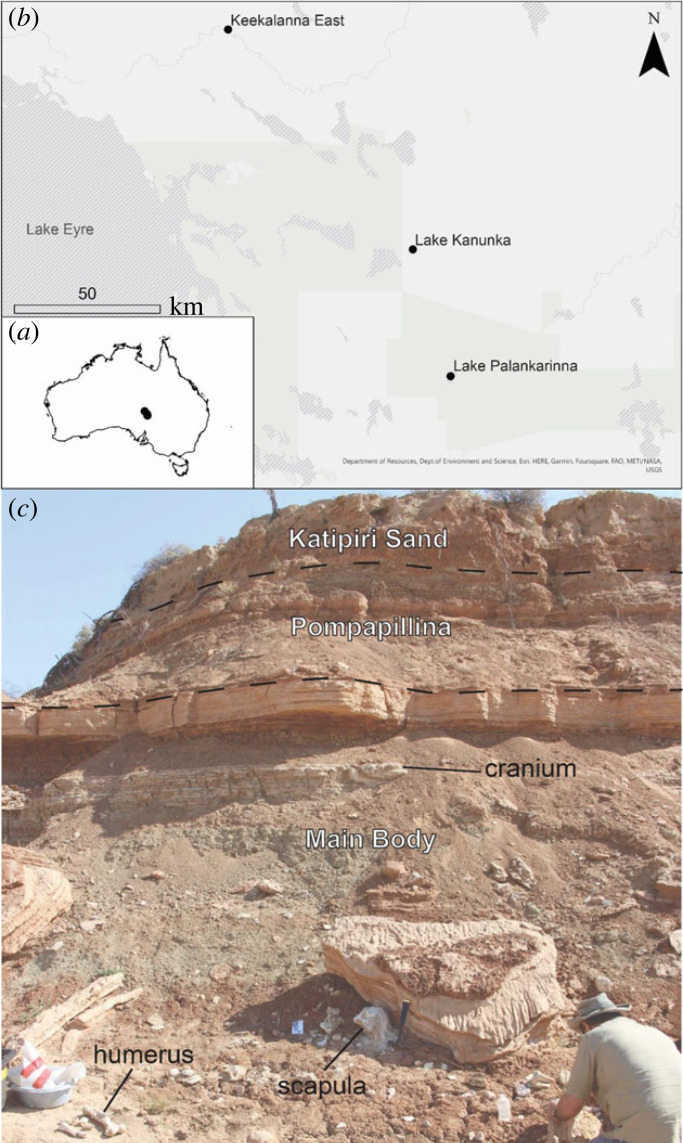
Map of fossil deposits where *Ambulator keanei* material was found (*a*,*b*) with Keekalanna East, Lake Kanunka and Lake Palankarinna. Close up of the Main body of the Tirari Formation as exposed at Keekalanna East with some elements *in situ* and stratigraphic context (*c*). Map created using World Topographic Map in ArcGis Pro. Sources: Department of Resources, Department of Environment and Science, Esri, HERE, Garmin, FAO, NOAA, USGS.

A partial skeleton of *Z. keanei* was collected by A.B.C. and T.H.W. in September 2017 from the Main Body of the Tirari Formation, exposed at the Keekalanna East Waterhole on the Warburton River, South Australia (figure 1). This specimen represents the first-known individual of a Pliocene diprotodontid composed of associated cranial and postcranial material. This has allowed the retrospective identification of remains of this species in collections from other sites where only partial cranial material had previously been identified. Comparisons of the partial skeleton of *Z. keanei* with those of *Z. trilobus* show numerous marked cranial and postcranial differences. Although the phylogeny of the Diprotodontidae is under re-assessment elsewhere and incorporates both dental and postcranial morphology, for the purposes of this work, it is suffice to note that these species express a level of distinction more commensurate with separation at the genus rather than species level.

The primary aims of this paper were to (i) provide a revised diagnosis, description and comparison of *Z. keanei*, (ii) describe the footpad impressions associated with the pes and (iii) make inferences about its functional morphology in relation to locomotion.

## 2. Methods

### 2.1. Nomenclature

Serial designation of the marsupial cheek dentition follows Stirton *et al*. [25] and Luckett [26]; upper case abbreviations designate upper dentitions (e.g. M1, I1); lower dentition is designated by lower case abbreviations (e.g. m1, i1).

Upper premolar cusp morphology follows Stirton *et al*. [25]. Some features of the lower premolar have often been left unnamed [9,11,14,25], which are given names here. Based on homology with the corresponding upper dentition [27], we refer to the central and posterior cuspids in Stirton *et al*. [25] and [9] as the protoconid and hypoconid as per [28]. In addition, we refer to the posterior ancillary crest in Stirton [10] as the ‘hypolophid’, because it unites the secondary cusp (hypoconid) with the tertiary cusp (entoconid) in diprotodontids, such as *Diprotodon optatum* and *Zygomaturus trilobus*, in which a postcingulid is found behind it.

Cusp terminology for the molars follows Stirton *et al*. [6], and Tedford & Woodburne [29], where the hypocone of the uppers is identified as the metaconule. In the Palorchestidae, stylar cusps C and D extend the protoloph and metaloph [30]. In diprotodontids, it is difficult to differentiate stylar cusp C and D from the paracone and metacone due to their incorporation into the lophs [9,30,31]. Consequently, studies refer to the buccal cusps of the protoloph and metaloph in diprotodontids either as stylar cups C and D [30,32] or as the paracone and metacone [9,11,30,33–35]. In addition, the anterobuccal and posterobuccal cusps may be referred to as stylar cusps B and E [30,32], parastyle and metastyle [8,9,34] or a combination of both [11,35]. A detailed review of molar cusp homology of the Diprotodontidae in relation to other members of the Vombatiformes is beyond the scope of this study. For now we follow Murray [9] and use the terms parastyle, paracone, metacone and metastyle for what may be stylar cusps B, C, D and E. The crista posterior to the paracone that is sometimes referred to as the postparaconal crest [6] or midlink [9] is here termed as the postparacrista as per [8]. We follow [30,36] in referring to the anterior portion of the cristid obliqua as the midlink.

The descriptions of postcranial material follow the *Nomina Anatomica Veterinaria* [37], supplemented by terms used in prior works on the group [16,34,38]. Muscle attachments were inferred based on Argot [39], Tschirn [40], Argot [41], Warburton & Marchal [42], Harvey & Warburton [43] and unpublished dissections of koalas and wombats carried out by J.D.v.Z. and A.B.C.

### 2.2. Collection

SAMA P54742 was collected from Keekalanna East, lower Warburton River, Kalamurina Station, South Australia, in the Main Body of the Tirari Formation by A.B.C. and T.H.W. in 2017. Permissions for fieldwork and fossil collection were granted by the Australian Wildlife Conservancy. The partially articulated skeleton had been eroded by the river when discovered but carbonate concretions preserved the skull and protected feet and other joints from further disarticulation. The material was prepared at the Flinders University Palaeontology laboratory and stabilized with Paraloid B-72. The near-complete, articulated pes was manually prepared out of its enclosing concretion with a micro-jack. Other material referred to *Z. keanei* and examined here was collected prior to this study [10,16].

Specimens of *Z. keanei* and comparative material were observed from the following institutions, with the most complete material digitized for direct comparisons: Flinders University, Adelaide (FU); Museum and Art Gallery of the Northern Territory, Alice Springs (NTM); Papua New Guinea National Museum and Art Gallery, Port Moresby (PNG); the South Australia Museum, Adelaide (SAMA); Queensland Museum, Brisbane (QM); Australian Museum, Sydney (AM); Museums Victoria, Melbourne (NMV); Queen Victoria Museum and Art Gallery, Launceston (QVM); Smithsonian Institution, Washington DC (USNM); University of California Museum of Palaeontology (UCMP); Natural History Museum, London (NHMUK); Tasmania Museums and Art Gallery (TMAG). Key specimens were digitized using an Einscan Pro + 3D surface scanner at between 0.24 mm and 0.7 mm resolution.

### 2.3. Segmentation of the pes

The near-complete pes of SAMA P54742 was scanned using computed tomography (CT) with a SOMATOM Force scanner by Dr Jones and Partners Medical Imaging at the South Australian Health and Medical Research Institute before the pes was completely prepared out of its concretion. Specimens were scanned with a 0.4 mm slice thickness to produce 2729 slices. The CT scans were imported into image processing software Mimics 22.0 where segmentation, thresholding and smoothing of the materials were carried out. The outline of the soft tissue was manually segmented at dorsal, lateral and anterior planes to ensure the accuracy of segmentation. The pedal elements were segmented using the ‘split mask’ function in Mimics v. 22. The matrix encasing the pes made it difficult for the X-rays of the CT scanner to penetrate and so high-resolution differentiation of the bone surfaces was not possible. Consequently, it was also manually prepared out of the concretion to allow the observation of the pedal morphology of the bones in detail. The structures resembling soft tissue within the matrix were not recognized in the scan images until after the bones were prepared and, as such, are no longer represented by a physical object.

### 2.4. Morphological comparison

All specimen comparisons were undertaken using a mixture of .stl files in the software programs 3-matic v. 14.0 and Blender 2.93, photos and observation of physical specimens. Three-dimensional meshes of specimens were aligned in the same plane/axis to enable consistent comparisons and then figured in orthographic view in the descriptions. All comparisons regarding proportions between taxa are relative to the element and not absolute unless stated otherwise. Comparisons were made with all the material listed in the electronic supplementary material. Comparisons were primarily restricted to taxa found in mainland Australia during the late Miocene to the Late Pleistocene with some exceptions mentioned in the text.

The late Miocene Alcoota Local Fauna contains four species of diprotodontid described on the basis of craniodental remains, but articulated or associated elements of individual skeletons are rare [44]. Nevertheless, Peter Murray, A.B.C. and J.D.v.Z. have made efforts to ascribe postcranial elements to species on size, relative abundance and morphological grounds and we use them for comparisons here.

We refer specimens formerly attributed to *Nototherium tasmanicum* Scott, 1911 [45] to *Zygomaturus trilobus*, which is in line with previous studies [38,47–49]. A formal re-assessment of *N. tasmanicum* is beyond the scope of this study.

The taxonomy of *Nototherium mitchelli* Owen, 1845 [50] and *Kukaodonta robusta* (De Vis, 1891) [51] is still uncertain and will be addressed in a forthcoming publication. Until then, we regard *Kukaodonta robusta* as a junior synonym of *Nototherium mitchelli* on the grounds that features displayed in the type of *Nototherium mitchelli* allow it to be distinguished from all other diprotodontids.

### 2.5. Body size estimates

Body size estimates of *Am. keanei* were made using the minimum humerus circumference using the equation in Richards *et al*. [38].

LogKg = (2.662*LOG(minimum humeral circumference mm)) − 0.108

Body size was also calculated using humeri from Alcoota diprotodontids for comparison with late Miocene taxa.

## 3. Results

### 3.1. Systematic palaeontology

Class: Mammalia Linnaeus, 1758 [52]

Superorder: Marsupialia Illiger, 1811 [53]

Order: Diprotodontia Owen, 1877 [54]

Superfamily: Diprotodontoidea Archer & Bartholomai, 1978 [55]

Family: Diprotodontidae Gill, 1872 [56]

Genus: *Ambulator* gen. nov.

**Zoobank registration**

ZooBank LSID of this publication: urn:lsid:zoobank.org:pub:949A693F-B15A-4552-AF51-CEB2B3299827

Zoobank LSID of genus: urn:lsid:zoobank.org:act:853C952C-66DB-4B8A-ADCC-1DCA3EFA9948

Type species: *Ambulator keanei* (Stirton, 1967)

Species: *Ambulator keanei* (Stirton, 1967) comb. nov.

#### 3.1.1. Genus etymology

‘Ambulator’ is Latin for ‘walker’. This is in reference to the taxon's many postcranial adaptations that to plantigrade, graviportal walking. The gender of the genus is masculine.

#### 3.1.2. Generic diagnosis

As for the species until another species is described.

#### 3.1.3. Synonyms

None

#### 3.1.4. Species etymology

Named for Mr Vincent P. Keane, who provided heavy equipment for the excavations in the Tirari Desert in 1962 conducted by Ruben A. Stirton, Paul F. Lawson, Richard H. Tedford and Michael O. Woodburne [10,57].

#### 3.1.5. Holotype

**SAMA P13844,** partial lower mandible with both ascending rami missing; right and left I1; left and right P3; left M1; right M4, partial maxilla containing left M2–M4. Collected by R. A. Stirton in 1962.

#### 3.1.6. Type locality

Keane Quarry, Lake Palankarinna, South Australia [10]. Palankarinna Local Fauna, 3.9 Ma.

#### 3.1.7. Paratypes

**UCMP 70121**, right M1 (Keane Quarry); **UCMP 70120**, right M2 (Keane Quarry); **UCMP 44622**, left I1 (Woodard Quarry); **UCMP 45409**, left I1 (Woodard Quarry). UCMP 44622 and UCMP 45409 were previously identified [10] as right I3s.

#### 3.1.8. Species diagnosis

*Ambulator keanei* is distinguished from all other diprotodontids by the following unique combination of traits.

The shape of the cranium is most similar to that of *Kolopsis torus* and differs by having an anterodorsally taller septum relative to the nasals.

The upper incisors differ from all other diprotodontids through the combination of the hypsodont, laterally diverging I1; a triangular I3 that is anteroposteriorly longer than the I2 and two dorsoventrally aligned buccal grooves on the I3. The P3 has five main cusps and is most similar to those of *Ma. ronaldi*, *Kolopsis yperus*, *Kolopsis rotundus* and *Z. trilobus*. The P3 differs from *Ma. ronaldi* by being similar in size to the M1. The P3 further differs from those of *Kolopsis yperus*, *Kolopsis rotundus* and *Z. trilobus* by the parastyle being slightly broader relative to the other cusps on the tooth. The upper molars can be confidently differentiated from all other diprotodontids except those of *Z. trilobus* and *H. tomasettii* by the absence of a forelink; a separated parastyle that is variably present on all molars; clear separation between the precingulum, lingual cingulum and postcingulum on all molars; a distinct mesostyle/buccal cingulum; low midlink; high postparacrista and postmetacrista; mesostyle partially connected to the postmetacrista; and a broad postmetaconulecrista.

The dentary can be differentiated from all other diprotodontids by having: a shallower symphysis than in those of *Euowenia grata* and *Pyramios alcootense*; a distinct digastric process positioned below the hypolophid of the m4; no clear separation of the digastric sulcus and the pterygoid fossa; the posterior masseteric eminence is positioned at the posteroventral edge of the masseteric fossa; and the coronoid process arises almost vertically above the mandibular corpus unlike that of *Z. trilobus*.

The i1 is most similar to those of *Meniscolophus mawsoni*, *Ma. ronaldi*, *Nototherium mitchelli* and *Z. trilobus*. The lower incisors can be further differentiated from those of *Z. trilobus* and *N. mitchelli* by being less dorsally curved. The p3 is most similar to those of *Z. trilobus*, *No. mitchelli, Kolopsis rotundus* and *M. ronaldi*. The protoconid on the p3 is broader relative to length than in that of *Z. trilobus*. The lower molars can be identified to the exclusion of all other diprotodontids except those of *Z. trilobus* and *H. tomasettii* by the combination of being lower crowned than those of *D. optatum* and *Eur. dunense*; wide, U-shaped interlophid valleys; the presence of the midlink and buccal cingulid; a low cristid obliqua; and low midlink.

The forelimbs of *Am. keanei* are most similar to those of *D. optatum* and *Z. trilobus*. The scapula lacks the large infraspinous process seen in that of *D. optatum* and is less medially curved than that of *Z. trilobus*. The deltopectoral crest of the humerus is straight, similar to that of *D. optatum* and unlike that of *Z. trilobus*. The supracondylar bridge is present, unlike in that of *D. optatum*.

The manus is most similar to those of *D. optatum* and *Z. trilobus,* differing by an hourglass-shaped pisiform*.* The triquetrum is smaller relative to the rest of the manus; the hamatal facet is mediolaterally narrow and angled almost parallel with the styloid facet. The styloid facet on the triquetrum is crescent shaped. A scaphoid facet is present on the hamatum but is not nearly as extensive relative to the hamatum in that of *D. optatum*. The hamate process is larger and projected more plantarly. The hamatal and scaphoidal facets on the capitatum converge to a point unlike in that of *Z. trilobus.* The radial facet on the scaphoid is concave similar to those of *Z. trilobus* and *D. optatum*. Unlike that of *D. optatum*, there is no differentiated hamatal facet that is larger than the capitatum facet on the scaphoid. The metacarpals are similar to those of *D. optatum* and unlike those of *Z. trilobus* with metacarpal 5 being the largest and triangular in dorsal view. The manual proximal and middle phalanges are smaller and taper more distally, and the manual ungual phalanges are narrower and taller than in *Z. trilobus*.

The hindlimb of *Am. keanei* is most similar to those of *D. optatum* and *Z. trilobus.* The lateral trochlear crest is larger than that of *Z. trilobus* and similar to that of *D. optatum*. The femoral condyles are asymmetrical with the medial condyle extending more caudally. There is no step in the lateral condyle such as those observed in *D. optatum* and *Z. trilobus.* There is no medial projection of the medial femoral facet on the tibia like in that of *D. optatum*. The femoral facet on the fibula is triangular and one-tenth the size of the tibial facet.

The pes is most similar to those of *Z. trilobus*, *Euo. grata* and *D. optatum*. The tibial facet on the talus is oval and takes up a smaller portion of the dorsal surface, unlike the larger rectangular tibial facet in those of *Z. trilobus*, *Euo. grata* and *D. optatum.* The calcaneal facet on the talus is more concave than in that of *Z. trilobus*, resulting in the posteromedial corner of the talus being deeper*.* The cuboid facet of the navicular differs from that of *Z. trilobus* by tapering dorsally to a thinner point, causing the facets for the talus and ectocuneiform to nearly meet. The cuboid is pentagonal in the dorsal aspect, similar to that of *Z. trilobus*. The medial plantar tubercle of the cuboid is larger than in that of *Z. trilobus* and is anteriorly hooked, similar to that of *D. optatum*. The mesocuneiform is fused with the entocuneiform but not with the ectocuneiform like in those of *Euo. grata*. Metatarsal 5 is triangular in dorsal view and curves medially, most similar to that of *Z. trilobus* and differs from those of *D. optatum* and *Euo. grata* by the lack of distinctly separate insertion points for the *mm. peroneus* on the lateral edge of metatarsal 5.

#### 3.1.9. Remarks

**SAMA P52377**, a partial right and left manus with radius and ulna and right and left pes, initially assigned to *Euowenia grata* [16]; **UCMP 70126**, right maxilla (preserving M2–M4). Stirton [10] identified this specimen as an unknown diprotodontid that was a different species from *Am. keanei*. As the premolar was not known, Stirton [10] did not designate it as a new species. Upon comparison with SAMA P54742, and in the light of variation observed in *Z. trilobus* specimens, we consider UCMP 70126 to be a variant of *Am. keanei;* **UCMP 70124**, right I3 previously identified as an I3 of *Me. mawsoni*, based on how it differs from the paratypes **UCMP 44622** and **UCMP 45409**. However, as there is no material to suggest *Me. mawsoni* is represented in Keane Quarry, we re-identify **UCMP 70124** as belonging to *Am. keanei* making the latter the only known diprotodontid taxon from the site; **UCMP 70131**, right I3 identified as *Am. keanei* for the same reasons as **UCMP 70124**.

#### 3.1.10. Referred material

***Woodard Quarry Lake Palankarinna, Tirari Formation, Mampuwordu member, approximately 3.9*** ***Ma*** [20,21]

**UCMP 44633**, partial left and right parietal; **UCMP 44626**, right calcaneus; **UCMP 44627**, right tibia; **UCMP 44401**, partial left dentary with m4 present.

***Lawson–Daily Quarry, Lake Palankarinna, Tirari Formation, Mampuwordu member, approximately 3.9 Ma***

**UCMP 70125**, highly worn, left I1; **UCMP 70126**, right maxilla with M2.

***Keane Quarry,* *Lake Palankarinna, Tirari Formation, Mampuwordu member, approximately 3.9 Ma***

**UCMP 71387**, partial maxilla with right incisors; right M2–M4, left M2 and M4, and left masseteric process; **UCMP 70121**, right M1; **UCMP 70123**, left p3, left m1; **UCMP 70120**, right M2; **UCMP 70124**, right I3; **UCMP 70131**, right I3.

***Mullet Locality, Lake Palankarinna, Tirari Formation, Mampuwordu member, approximately 3.9 Ma***

**UCMP 297918**, left maxilla, right maxilla with right M3–M4, right P3, right M1–M2, right I1, left and right I1, a partial epitympanic; **UCMP 322914**, mandible.

***Keekalanna East, Warburton River, Tirari Formation, Main Body, 3.6–3.9 Ma*** [20,21]

**SAMA P54742**, partial skeleton consisting of a cranium, left dentary, atlas, axis, three other cervical vertebrae, four thoracic vertebrae, right clavicle, manubrium, rib fragments, right and left scapula, left humerus, partial right humerus, partial left and right radius, distal left ulna, complete left manus (consisting of a pisiform, triquetrum, hamatum, capitatum, scaphoid, trapezium, metacarpals 2–5, ungual phalanges 1–5; a middle phalanx; two proximal phalanges), left femur, left tibia, left fibula and near-complete left pes, with ungual phalanges 2 and 3 missing; **SAMA P52377**, partial left and right articulated manus with distal radii and ulnae attached, right manus (missing trapezium, trapezoid and ungual phalanx 5), left manus (missing the trapezoid), a fragmentary distal right tibia, the left pes (with a partial calcaneus, entocuneiform, entocuneiform, cuboid, talus and metatarsal 5), a right pes (with partial/fragmentary talus, navicular, calcaneus, cuboid, entocuneiform, metatarsal 5 and proximal phalanx 5).

***Lake Kanunka, Tirari Formation, Pompapillina Member, 3.6 Ma*** [20,21]

**UCMP 66917**, right M4.

### 3.2. Descriptions and comparisons

#### 3.2.1. Cranium

**Figure 2.**
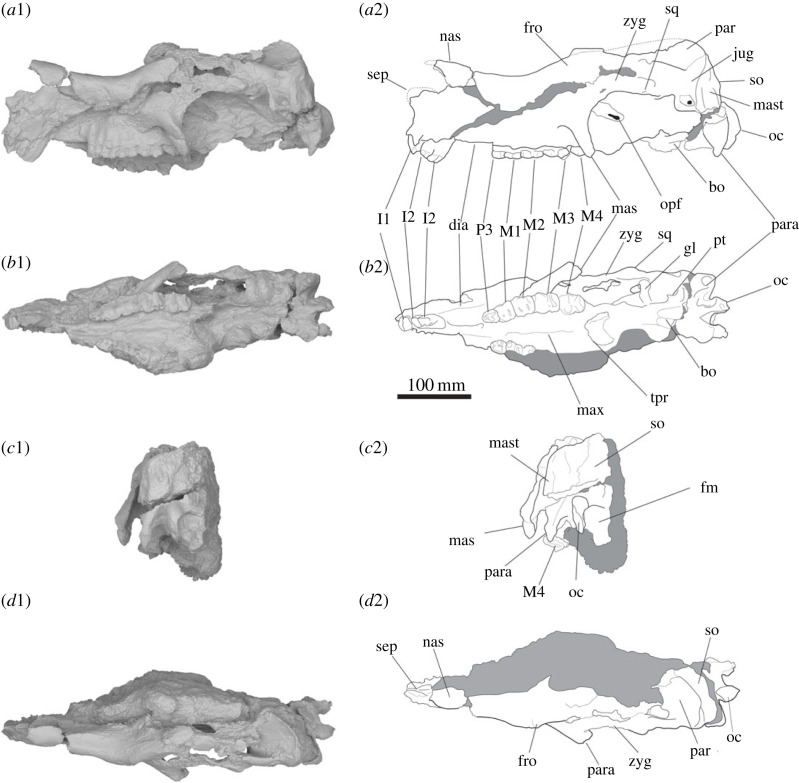
Surface scans (1) and illustrations (2) of the cranium of *Ambulator keanei* (SAMA P54742). Lateral view (*a*), ventral view (*b*), posterior view (*c*) and dorsal view (*d*). Abbreviations: **bo** basioccipital crest; **dia**, diastema; **fro**, frontals; **gl**, glenoid fossa; **jug**, jugal; **mas**, masseteric process; **mast**, mastoid; **max**, maxilla; **nas**, nasals; **so**, supraoccipital; **oc**, occipital condyle; **fm**, foramen magnum; **opf**, optic foramen; **orb**, orbit; **par**, parietals; **para**, paroccipital process; **pt**, pterygoid; **sep**, septum; **sq**, squamosal; **tpr**, transverse palatine foramen; **zyg**, zygomatic arch. Shaded areas denoted regions that are damaged or concealed by sedimentary matrix.

SAMA P54742 (figure 2) is the most complete cranial specimen known for *Ambulator keanei*. It consists of an adult cranium, although the right half and most of the basicranium are damaged or missing. The cranium is anteroposteriorly elongate (even allowing for post-depositional distortion), tapering anteriorly over the length of the rostrum in dorsal view. A bony septum arises from the premaxilla, projecting anterodorsally from above the I1. Although damaged, it appears to have been triangular in lateral view. The nasals extend dorsal of the incisors and have a concave anterior border in lateral view. In dorsal view, the nasals terminate anteriorly in a sharp medial tip above the septum, and broaden posteriorly, with an anteriorly concave margin, to meet the edge of the frontals above the orbits. The diastema is 52.5 mm long, with the palatal surface of the premaxilla being considerably narrower than that of the maxilla.

In dorsal view, the frontals project laterally above the orbit before tapering anteriorly towards the nasals. The frontals are arched slightly dorsally when viewed from the lateral aspect. The left parietal plate is present, but only the posterior section adjacent to the occiput remains. The parietal plate is convex in vertical cross-section and meets medially with the other plate to form a weak sagittal crest. The maxilla is slightly convex at the buccal margin, tapering anteriorly towards the premaxilla. The orbit is crushed and slightly distorted, and situated below the border of the nasofrontal suture. The transverse palatine foramen is strongly concave with the apex level with the protoloph of the M4. The anterior half of the pterygoid cavity is present in SAMA P54742; it is located below the optic foramen, tapers anteriorly, and the surface is buccally concave. The anterior half of the basioccipital crest is present; it is located posterior to the pterygoid cavity with strong oval depressions on buccal sides. A deep, transverse canal extends from the posterior face of the orbit along the maxilla to a large foramen that presumably includes the optic foramen and the rostral alar canal. The masseteric process extends ventrally to be level with the molar occlusal surface and terminates level with the interloph valley of the M4. The zygomatic arch is arched dorsally in lateral view. The glenoid fossa is flat and located at the base of the jugal. Most of the basicranium is cracked and slightly distorted, obscuring many of the sutures. The posterior epitympanic fossa is located between the postglenoid process and the mastoid. The paroccipital process is directly posterior to the epitympanic fossa and is buccolingually compressed and slightly hooked anteriorly. The paroccipital process tapers ventrally, before terminating level with the tooth row. The mastoid is confined to the occiput and is angled slightly anteriorly to the rest of the occiput. The occipital condyles are dorsoventrally elongate, having near-equal depth to the paroccipital process. They taper slightly ventrally and have a convex articular surface. The foramen magnum is oval and slightly dorsoventrally compressed.

Overall, the shape of the cranium is more similar to that of *Kolopsis torus*, with a relatively shallow zygomatic arch and masseteric process, than to those of *Zygomaturus trilobus* and *Maokopia ronaldi.* The prominent septum is similar to that observed in *Z. trilobus* and *Diprotodon optatum*, and is unlike the smaller/absent bony septum in *Neohelos stirtoni*, *Kolopsis torus*, *Plaisiodon centralis* and *Pyramios alcootense*. In contrast with those of *D. optatum*, no nasal fossa is present in *Am. keanei*, which is similar to that seen in *Z. trilobus.* The nasals are positioned close to the incisor arcade, which is more similar to the state in *Kolopsis torus* and *Pl. centralis* and unlike the extremely posteriorly positioned nasals in *Euowenia grata*. The nasals of *Am. keanei* are not as laterally flared as those of *Z. trilobus*. The masseteric process is similar to that of *Kolopsis torus*, as opposed to the more extensive and anteroposteriorly flattened masseteric process observed in those of *Euo. grata*, *Euryzygoma dunense* and *Z. trilobus*. The orbit faces more laterally than in those of *Z. trilobus* and *Ma. ronaldi*. The frontals arise less steeply above the nasals than in those of *Z. trilobus*, *Ma. ronaldi* and *Hulitherium tomasettii*. The basicranium is aligned close to level with the tooth row, similar to that seen in those of *Kolopsis torus* and *Pl. centralis*, but unlike in those of *Eur. dunense*, *D. optatum* and the more extreme *Z. trilobus,* which are more dorsally angled relative to the tooth row. The occiput is more similar in both size and angle to that of *Kolopsis torus* than the broad, anteriorly oriented occiput of *Z. trilobus*. The paroccipital process is more similar in shape and size to that of *Pl. centralis* (NTM P7333) than that of any other diprotodontid.

#### 3.2.2. Upper dentition

The I1 is anteroventrally curved and tapered in buccal view. Enamel of the I1 is restricted to the buccal side. The I2 is the smallest incisor and is circular in cross-section. There are two dorsoventrally aligned grooves on the buccal side of I3, as observed in UCMP 70124, that form a single prominent groove when worn (UCMP 70131). The triangular occlusal outline on the relatively unworn I3 in UCMP 70124 and UCMP 70131 becomes square with wear, as seen in SAMA P54742 (figures 5 and 6).

The I1 of *Am. keanei* is identical to that of *Z. trilobus*. The I2 of SAMA P54742 is too worn for adequate comparison but is larger relative to the I1 than that of *Z. trilobus*. The I2 and I3 are larger in proportion to the I1 than in those of *Z. trilobus* (observable between SAMA P54742 and QVM1990GFV115). The triangular I3 (SAMA P13844) is most similar to those of *Eur. dunense* and *Alkwertatherium webbi*. The incisors of *Am. keanei* differ from *Euo. grata* by the presence of the I3; a proportionately larger I2 and the enamel being more restricted to the buccal side of the I1. The incisors of *Am. keanei* differ from those of *D. optatum* by the I1 being rooted (i.e. not hypselodont) and splayed, tapering to a point; and the occlusal surface of the I3 is triangular with a buccal groove, as opposed to the rounded occlusal surface in that of *D. optatum*. The incisors of *Am. keanei* differ from those of *Kolopsis torus*, *Py. alcootense*, *Al. webbi* and *Pl. centralis* in that the I1s are larger relative to the I2 and I3; are anteroventrally curved; and tapered to a point.

The P3 has a roughly pentagonal occlusal outline, has five cusps and is slightly smaller than the M1 (table 1). The length of P3 relative to width varies between all specimens with SAMA P54742 being the most elongate and SAMUC388b being the least elongate (table 1). The paracone is the largest and tallest cusp observable in SAMUC388b, followed by the metacone, parastyle, protocone and hypocone. The parastyle is situated at the most anterior point on the tooth and hooks slightly posteriorly. The protocone is situated buccal to the paracone, with a low cingulum extending between the buccal extremities of both. The paracone and protocone are either partially connected at their base (SAMA P13844 and SAMA P54742) or separated by a valley (UCMP 297918 and UCMP 70122). The metacone is directly posterior to the paracone and both are partially connected to form a parametacone (figure 2). A groove on the buccal side of the junction of the paracone and metacone varies from shallow in SAMA P54742 to deep in UCMP 70122. A mesostyle is variably present at the base of this buccal groove and varies from distinct (SAMA P13844) to weak/absent (UCMP 297918, UCMP 70122, SAMA P54742). The protocone is directly lingual to the paracone with both cusps partially connected at their bases. The hypocone is directly behind the protocone and varies in relative size from weakly developed (UCMP 297918) to well developed (SAMA P13844). The base of the hypocone occasionally makes contact with the base of the protocone (SAMA P13844) and with the metacone (UCMP 297918). A central basin is formed by the arrangement of the metacone, hypocone, protocone and paracone. A shallow lingual cingulum extends between the posterolingual base of the protocone and the anterolingual base of the hypocone. A posterior cingulum extends between the posterior base of the hypocone and metacone.

**Table 1.** Upper tooth row dimensions in millimetres for all specimens of *Ambulator keanei.* Abbreviations: **L**, length; **AW**, anterior width; **PW**, posterior width.

specimen	side	P3L	P3PW	M1L	M1AW	M1PW	M2L	M2AW	M2PW	M3L	M3AW	M3PW	M4L	M4AW	M4PW
SAMA P13844	left	24.1	20.5	32.5	—	25.5	34.5	30.2	27.6	38.5	34.6	30.6	41.5	33.5	27.6
SAMA P13844	right	24.6	20.4	—	—	—	—	—	—	—	—	—	—	—	—
UCMP 44398	left	—	—	32.5	27.2	27.4	38.7	33	30.8	42.4	35.6	31.7	—	—	—
UCMP 44400	left	—	—	27.1	25.2	25.4	—	—	—	—	—	—	—	—	—
UCMP 44623	left	—	—	—	—	—	36.9	—	31.3	—	—	—	42.4	35.5	—
UCMP 45354	right	—	—	—	—	—	—	—	—	—	—	—	39	32	25.2
UCMP 54742	left	20.8	—	24.6	24	25.1	29.9	27.1	25	32.7	29.1	24.4	33.5	28.9	23.5
UCMP 54742	right	22.4	17.4	—	—	—	—	—	—	—	—	—	—	—	—
UCMP 70120	right	—	—	—	—	—	33.8	29.6	27.3	—	—	—	—	—	—
UCMP 70121	right	—	—	31	26.7	26.9	—	—	—	—	—	—	—	—	—
UCMP 70126	right	—	—	—	—	—	28.9	26.3	25.1	33.8	29.1	25.8	34.2	28.1	22.2
UCMP 71387	left	—	—	—	—	—	35	30.5	27.5	—	—	—	39.8	32.7	25.8
UCMP 71387	right	—	—	—	—	—	—	—	29.6	39.1	32.2	29.2	40.7	33.1	26.2
UCMP 297918	left	26.7	19.7	32.3	29.7	28.9	39.9	32.1	33.4	45.2	33.7	31.7	—	35.5	—
UCMP 297918	right	25.8	20.6	32.1	28.3	28.2	—	31.8	—	—	36.2	32.4	39.8	34.9	27.5
UCMP 66917	left	—	—	—	—	—	—	—	—	—	—	—	39.2	33.4	26.3

**Figure 3.**
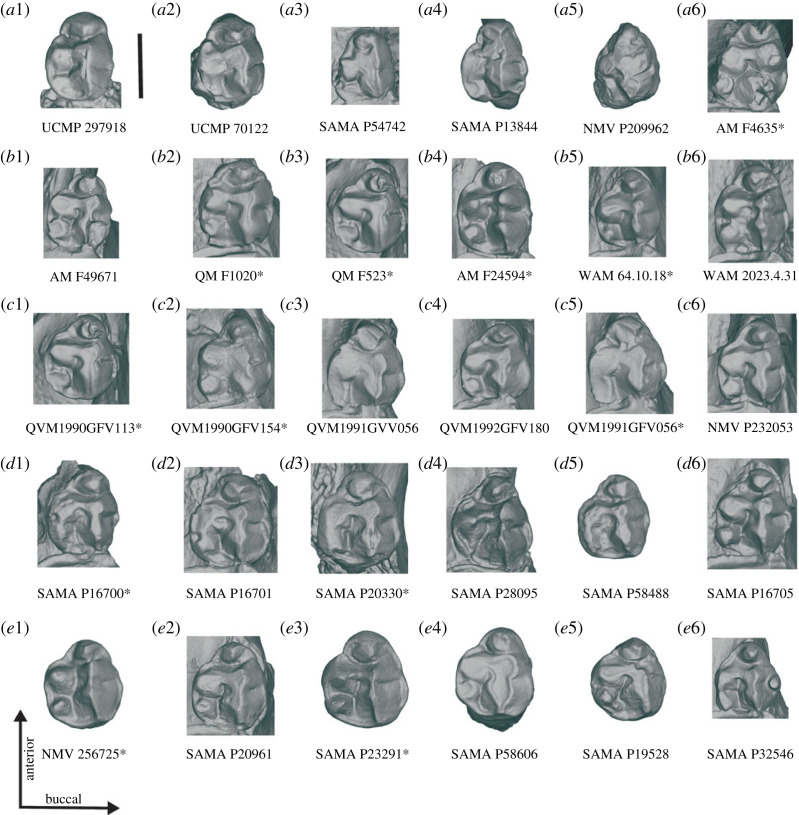
Comparisons of the left P3 of *Ambulator* *keanei* (*a*1–*a*4)*, Z. gilli* (*a*5) and *Z. trilobus* (*a*6–*e*6). Holotypes (*a*4, *a*5 and *a*6). Fossil localities: Mullet Locality (*a*1), Woodard Quarry (*a*2), Keekalanna East (*a*3), Keane Quarry (*a*4), Darling Downs (*a*6–*b*1), Borenore (*b*2–*b*3), Bingara (*b*4), Mammoth Cave (*b*5–*b*6), Mowbray Swamp (*c*1–*c*5), Sorrento Back Beach (*c*6), Victoria Fossil Cave (*d*1–*d*6), McEachern's Cave (*e*1), Warburton River (*e*2), Henschke's Quarry Cave (*e*3,*e*4) and Black Creek Swamp (*e*5,*e*6). Scale bar, 10 mm. Right specimens are mirrored (*) to allow easier comparisons.

The P3 of *Ambulator keanei* is most similar to those of *Z. trilobus* and *Z. gilli*. However, the morphology of the P3 is quite variable (figure 3). The P3 of *Am. keanei* can be differentiated from those of *Z. trilobus* by the slightly broader parastyle relative to the other cusps and the inconsistent partial linkage of the parastyle and paracone (SAMA P13844 and SAMA P54742). The buccal groove in the parametacone is slightly shallower in that of *Z. gilli* (NMV P209962) but this seems to be highly variable in both *Am. keanei* and *Z. trilobus,* with the variation in *Z. trilobus* encompassing what is observed in those of *Am. keanei* and *Z. gilli* (figure 3). The P3 in the holotype of *Z. gilli* is too damaged for most measurements to be reliable and it cannot be distinguished from the P3 of *Z. trilobus* (figure 3).

**Figure 4.**
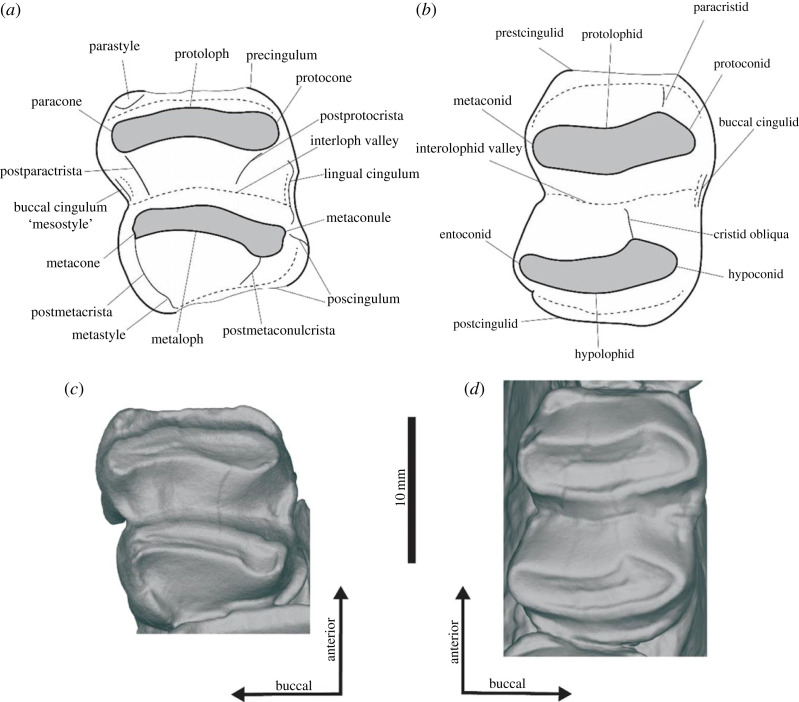
Key features of the right m2 (SAMA P13844) (*a*,*c*) and right M2 (UCMP 70126) of *Ambulator keanei.* Illustration of features with labels (*a*,*b*) and surface scans (*c*,*d*) of m2 and M2, respectively. The exposed dentine in the illustration is filled in with grey and enamel left white.

The upper molars are bilophodont, wide and sub-rectangular in occlusal view. Their size increases from M1 to M3, but M4 is smaller due to its narrower metaloph (table 1). Molar size varies between specimens, with the largest observed in UCMP 297918 and the smallest in UCMP 70126 (table 1). When viewed occlusally, the protoloph (anterior) and metaloph (posterior) are posteriorly concave. When viewed lingually, both the protoloph and metaloph slope anteriorly and are low crowned with a broad, U-shaped interloph valley separating the two. The protoloph and metaloph have subequal widths in all molars except M4. The precingulum is large, forming a low, distinct shelf extending from the anterior of the protocone to the anterior of the paracone in all molars. The parastyle is anterior to the paracone on the buccal edge of the precingulum on all molars. The paracone becomes proportionally smaller and less distinct from M1 to M4, and is absent/indistinguishable from the precingulum on the M4 of some specimens (UCMP 45354 and UCMP 297918). No preparacrista is observed on any of the molars. A mesostyle is present at the posterior base of the protocone in UCMP 44398, UCMP 66917 and the M2 of UCMP 71387. The expression of the mesostyle is variable both between and within specimens, with some (UCMP 70126, the M4 of UCMP 71387 and SAMA P13844) having the buccal cingulum restricted to the anterior half of the interloph valley. The postparacrista forms a distinct convexity at the base of the posterior protoloph in the buccal quarter of the interloph valley (UCMP 71387, UCMP 44398, UCMP 297918, UCMP 45353 and SAMA P13844) (figure 4). In UCMP 70126, SAMA P54742 and UCMP 66917, the postparacrista joins with the posterobuccal edge of the paracone midway up the protoloph and kinks buccally at the intersection. In UCMP 70126, this is most apparent in the M3 and M4, with the M2 being more similar to other specimens. On the M3 of UCMP 70126 but no other specimen, a faint secondary crest extends along buccal of the postparacrista. There is a faint convexity of the postmetaconule at the base of the metaconule posterior to the postparacrista in the M2 and M3 of UCMP 44398, M2 of UCMP 70120 and M1 of UCMP 70121, but this is not visible in other specimens. A postprotocrista forms a large convexity on the posterior face of the protoloph and extends from the apex of the protocone to the lingual third of the interloph valley, where it merges into the protoloph. A lingual cingulum extends from the posterior base of the protocone to the anterior base of the metaconule. The lingual cingulum is tallest on the M1 and is shallower on each successive molar. A bump is present on the posterior base of the protocone in some specimens (UCMP 44298, UCMP 13844, UCMP 71387, SAMA P13844 and UCMP 70120) but not in others (UCMP 70126, UCMP 66917, UCMP 44400 and UCMP 297918). A similar but weaker bump can be observed on the anterior base of the metaconule on the M2 of UCMP 44398. Since the relationship of these convexities is unknown, we refer to these features as the postprotoconule crest and premetaconule crest, respectively, in later comparisons. A strong postmetacrista is present on all molars and extends from the apex of the metacone, down the buccal edge of the metaloph to meet with the metastyle and the postcingulum. The postmetacrista is the tallest and most distinct in the M1, becomes progressively shallower on posterior molars and is weakest on the M4 to the point of being near-absent in some specimens (UCMP 44398, SAMA P13844). The mesostyle is a small convexity posterior to the metaconule on the buccal edge of the postcingulid. Like the metaconule, the mesostyle is tallest and most distinct on the M1 and is progressively weaker posteriorly, absent on the M4 of all specimens. The postmetaconulecrista is a large convexity on the posterior surface of the metalophid, originating from the apex of the metaconule and extending to the midline of the base of the metaloph. The postcingulum extends across the entire posterior surface of the metaloph from the metastyle, up the lingual edge of the metaconule, nearly contacting the lingual cingulum in the M1–M3. In the M4, the postcingulum terminates at the posterior base of the metaconule.

The upper molars of *Am. keanei* are most similar to those of *Z. trilobus*, *H. tomasettii* and NMV P194569 (previously referred to *Z. gilli*). Given the range of intraspecific variation observed within the upper molars of *Am. keanei* and other taxa, it is difficult to find definitive features that differentiate *Am. keanei* from those of *Z. trilobus*, *H. tomasettii* and NMV P194569*.* However, the presence of the buccal cingulum/mesostyle differentiates *Am. keanei* from NMV P194569. This cannot differentiate *Am. keanei* from *H. tomasettii*, as in the latter, the cingulum is variable; the buccal cingulum is absent in the M2 of PNG MR25077 and present in the M4 of PNG MR25191. There is also overlap with some specimens of *Z. trilobus* (e.g. AM F4635, AM F24594, QVM1990GFV154 and SAMA P32546) which have a distinct buccal cingulum, though other specimens of *Z. trilobus* lack any buccal cingulum (e.g. QMV1992GV180, WAM64.10.18). The postprotocrista appears to be larger and more distinct than that of *Z. trilobus* and broader than that of NMV P194569. The variable presence of the larger kinked postparacrista observed in UCMP 70126, SAMA P54742 and UCMP 6617 can occasionally be used to distinguish *Am. keanei* from those of *Z. trilobus, H. tomasettii* and NMV P194569.

#### 3.2.3. Mandible

**Figure 5.**
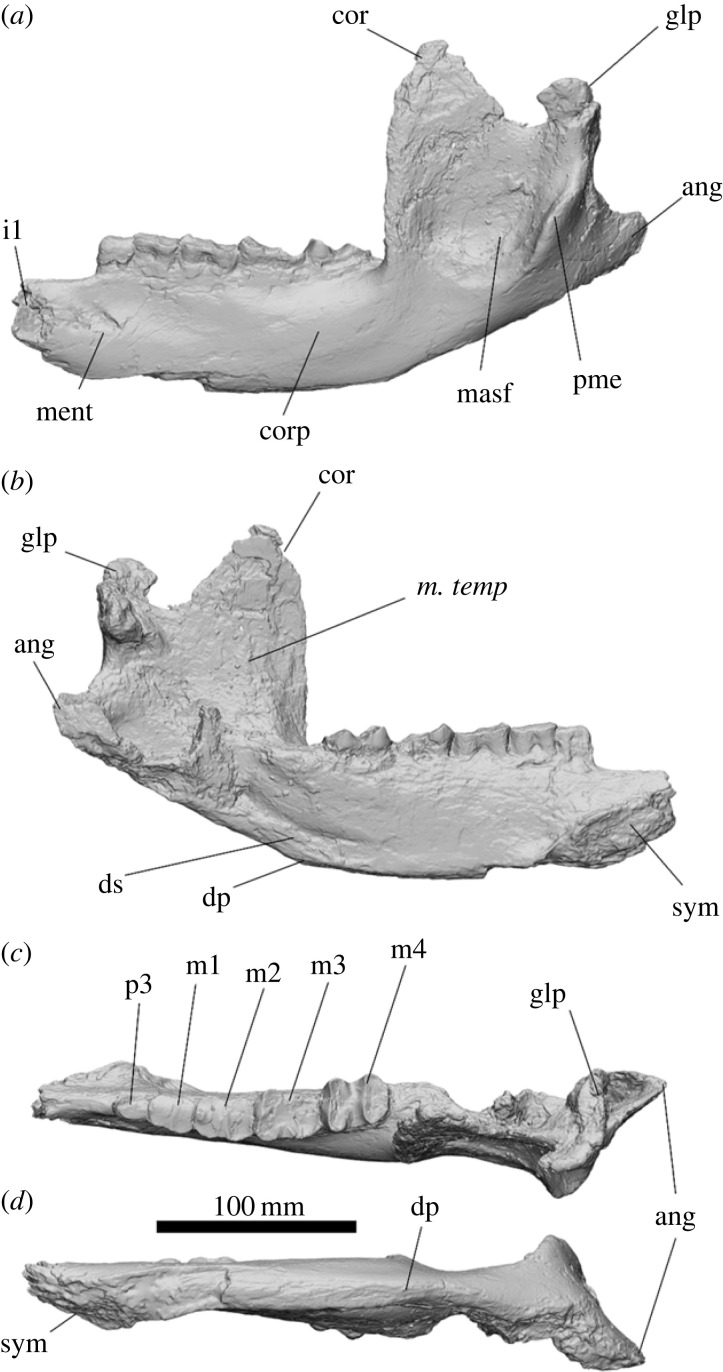
Left dentary of *Ambulator keanei* (SAMA P54742). Buccal view (*a*), lingual view (*b*), occlusal view (*c*), ventral view (*d*). Abbreviations: **ang**, angular process; **cor**, coronoid process; **corp**, mandibular corpus; **dp**, digastric process; **ds**, digastric sulcus; **glp**, glenoid process; **masf**, masseteric fossa; **ment**, mental foramen; **pme**, posterior masseteric eminence; **sym**, symphysis.

The mandibular corpus is high and narrow, with both the tooth row and ventral margins straight and level. The symphysis is oval and anteroposteriorly elongate, extending below the posterior extremity of the p3. The diastema is narrow and straight, comprising approximately 13% of the dentary length. The mental foramen is positioned below the anterior extremity of p3; a pathology obscures the foramen in SAMA P54742. The digastric sulcus is long and deep, extending from below the coronoid process to below the m3. The digastric process is abraded but appears to be positioned directly below the hypolophid of the m4. No clear separation between the pterygoid fossa and the digastric sulcus was observed (figure 5). The angular process is large and posterolingually flared with a deep pterygoid fossa. The coronoid process rises above the glenoid process; the anterior border is nearly vertical for the first two-thirds before gently curving posteriorly to the apex. The masseteric fossa is a large shallow depression on the buccal side of the ascending ramus; it originates level with the tooth row where it is deepest; a large posterior masseteric eminence is present on the posterior border. The glenoid process is partially damaged in SAMA P54742 but is widest on the buccal edge and tapers lingually.

The symphysis is shallow and slightly dorsally inflected, similar to those of *Kolopsis torus*, *Pl. centralis, Eur. dunense* and *Z. trilobus*; this is unlike the deeper symphysis in those of *Euo. grata*, *Me. mawsoni*, *Py. alcootense* and *D. optatum.* The digastric process, while most similar in size and position to that of *Z. trilobus*, differs by being slightly weaker and positioned closer to the tooth row. The digastric process is larger and more pointed than in that of *Kolopsis torus.* No clear separation of the digastric sulcus and pterygoid fossa was observed in *Am. keanei*, *Kolopsis torus*, *Py. alcootense*, *Pl. centralis, Me. mawsoni* or *D. optatum.* The coronoid process is less anteriorly angled than in that of *Z. trilobus* (NMVP 232053). The posterior masseteric eminence is positioned at the base of the masseteric fossa, unlike in those of *D. optatum* and *Euo. grata*.

#### 3.2.4. Lower dentition

The i1s diverge anterobuccally, and curve dorsally. The i1 in cross-section is taller than its width, with enamel mostly confined to the ventral surface and the ventral half of the lateral surface.

The i1 of *Am. keanei* is most similar to those of *Z. trilobus*, *Me. mawsoni* and *Nototherium mitchelli* and can only be differentiated from *Ma. ronaldi* by its larger absolute size. The I1 is less dorsally curved than those of *Z. trilobus* and *No. mitchelli*.

**Figure 6.**
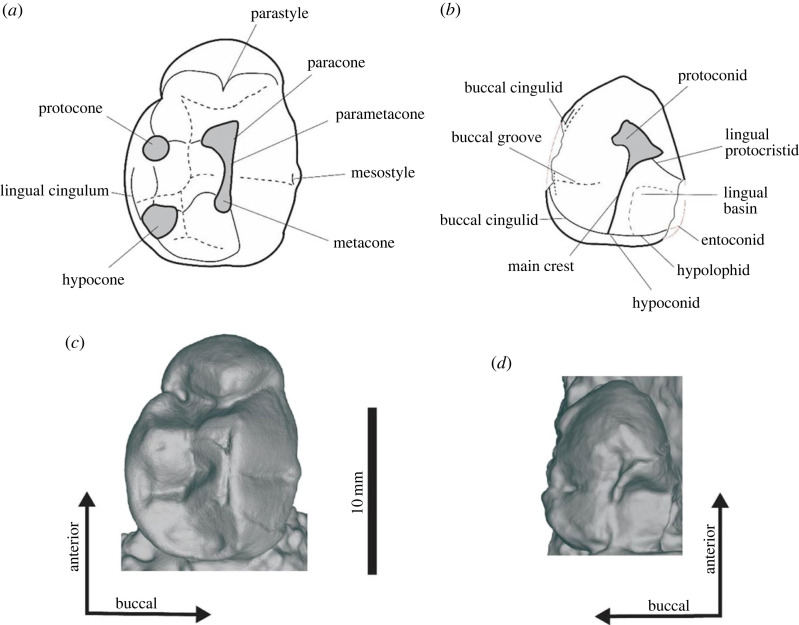
Key features of the left P3 (UCMP 297918) (*a*,*c*) and left p3 (UCMP 322914) (*b*,*d*) and of *Ambulator keanei.* Illustration of features with labels (*a*,*b*) and surface scan images (*c*,*d*). Dotted red lines are inferred morphology based on UCMP 70123.

The p3 is triangular and anterodorsally elongated in occlusal view (figure 6). The length of p3 relative to width varies between all specimens with SAMA P54742 being the most elongate and UCMP 297918 being the least elongate (table 2). The protoconid is large and makes up the entire anterior half of the tooth. A low lingual protocristid tapers posterolingually to the lingual edge of the tooth. The protoconid and hypoconid are connected by the blade-like main crest. The hypolophid extends from the hypoconid, at the midline of the tooth, to the entoconid on the anterior border of the tooth in UCMP 70123. A distinct buccal cingulid extends along the entire length of the tooth and up the buccal face of the hypoconid. A small lingual basin is present between the main crest, the lingual protoconid and the hypolophid. Due to compression/abrasion of the posterior edge by the m1 in all p3s observed, we are unable to determine the presence or absence of a postcingulid.

**Table 2.** Lower tooth row dimensions in millimetres for all specimens of *Ambulator keanei.* Abbreviations: **L**, length; **AW**, anterior width; **PW**, posterior width.

specimen	side	P3L	P3PW	M1L	M1AW	M1PW	M2L	M2AW	M2PW	M3L	M3AW	M3PW	M4L	M4AW	M4PW
SAMA P13844	right	18	15.3	—	19.2	—	34	25.4	25.1	39.5	28.1	28.7	43.1	30.6	27.9
SAMA P54742	left	18.5	11.37	23.1	16.02	17.8	29.2	21.3	22.0	34.5	26.3	—	—	—	—
UCMP 322914	right	20.3	—	—	—	—	—	—	34.6	45	35.3	33.8	49.7	36	31.8
UCMP 322914	left	20.4	16	34.1	23.6	27.4	43	30.4	29.5	44.5	32.8	31.3	46.7	32.5	—
UCMP 70123	left	20.5	15.5	29.6	19.5	22.2	—	—	—	—	—	—	—	—	—
UCMP 44399	left	—	—	37.9	—	28.3	42.3	32	28.7	43.1	32.3	28.0	—	—	—
UCMP 44401	left	—	—	—	—	—	—	—	—	—	—	—	43.5	31.9	29.3
SAMA P54742	right	—	—	—	—	—	—	—	—	—	—	—	34.7	26.8	25.3

The p3 of *Am. keanei* is most similar to those of *Z. trilobus, Ma. ronaldi*, *Kolopsis rotundus* and *No. mitchelli*. It differs from *Ma. ronaldi* by the presence of the buccal cingulum, similar to those of *Ma. ronaldi* and *No. mitchelli*. It differs from *Z. trilobus* by having a more bulbous protoconid in proportion to the length of the tooth.

All molars are rectangular and anteroposteriorly elongate in occlusal view. When viewed in occlusal aspect, all lophids slope posteriorly, are bowed such that they are anteriorly concave; low crowned with broad U-shaped interlophid valleys in buccal view. Molar size varies between specimens, with the smallest being SAMA P54742 and the largest being SAMA P22914 (table 2). A low precingulid is present on all molars; this is separated into two parts on the m1 by the paracristid, forming an anterobuccal basin that extends lower down the protolophid than the precingulid, lingual to the paracristid. A discrete paraconid is absent in all molars and is instead fused with the paracristid. The paracristid is tallest and most distinct on the m1 and lowest on the m2–m4, and terminates at the precingulid in the centre of the tooth on the m1 and posterior to the precingulid on the buccal quarter of the m2–m4. A low buccal cingulid is present in the interlophid valley, which extends up the posterior of the protoconid and the anterior face of the hypoconid. A low lingual cingulid is also present within the interlophid valley. A low midlink is present in the interlophid valley at the centre of the base of the protolophid that contacts the cristid obliqua. The expression of the midlink varies from being present in all molars (SAMA P13844), to being absent in all molars (UCMP 44399). A distinct cristid obliqua is present on all molars and terminates along the midline of the tooth in the centre of the interlophid valley. The postcingulid forms a low shelf, extending across most of the posterior face of the hypolophid, originating on the posterobuccal corner of the hypoconid and terminates posteriorly of the entoconid.

The lower molars of *Am. keanei* are most similar to those of *Z. trilobus* and *H. tomasettii,* differing only by the postcingulid being slightly less posteriorly extended, the paracristid terminating in the midline of the m1 as opposed to in the buccal quarter, and having a larger anterobuccal basin on the m1.

#### 3.2.5. Vertebrae

##### 3.2.5.1. Atlas (C1)

**Figure 7.**
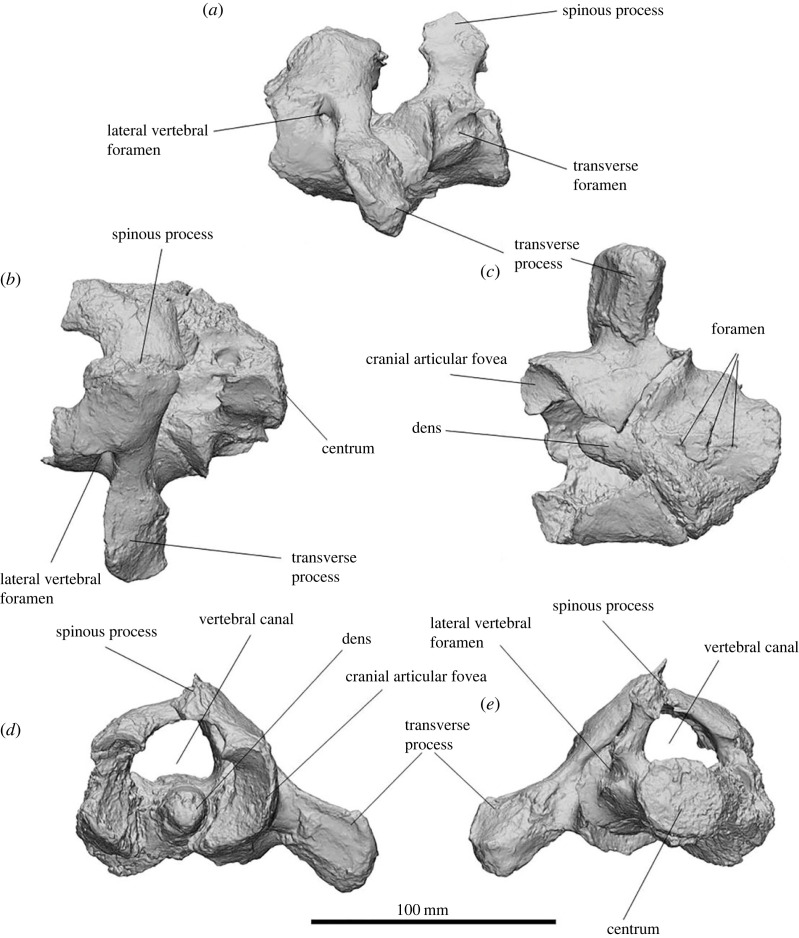
Atlas and axis of *Ambulator keanei* (SAMA P54742). Lateral view (*a*), dorsal view (*b*), ventral view (*c*), cranial view of atlas (*d*) and caudal view of axis (*e*).

The atlas and axis of SAMA P54742 could not be separated from each other during preparation without risking further damage (figure 7). The cranial articular fovea is crescent shaped from the cranial aspect, being 47.4 mm deep and 27.7 mm wide, with the lateral side extending cranially and sloping caudomedially. The transverse processes extend laterally 55 mm from the centrum and are angled slightly ventrally, with large attachment areas for *m. longissimus dorsi*, *m. obliquus capitis caudalis* and *m. obliquus capitis cranialis*. The lateral vertebral foramen is 9.5 mm wide and positioned just dorsolateral to the centrum. The spinous process (dorsal tubercle) is small, forming a slight raised surface on the dorsal centrum. The ventral arch is unfused but converges on the midline. The caudal articular fovea is obscured by the axis in SAMA P54742, but in UCMP 297918, it is less concave than the cranial articular fovea and more dorsoventrally compressed.

The cranial articular fovea differs from that in *Z. trilobus* (e.g. WAM 96.3.386) by tapering slightly anteriorly. The transverse foramina are mediolaterally narrower than in those of *Eur. dunense* (SAMA P23346), with a deeper and more oval cranial articular surface and narrower transverse processes. The transverse processes are mediolaterally narrower relative to length than in those of *D. optatum* (UCMP 60870). The atlas of *Am. keanei* lacks the large caudal tubercles on the ventral processes seen in those of *Z. trilobus* (WAM 96.3.386).

##### 3.2.5.2. Axis (C2)

The axis of SAMA P54742 is robust (84.5 mm long, max. width 81.4 mm) and is arrow shaped in ventral view. The cranial articular surfaces of the axis are oval and concave to mirror the convex articular facets on the atlas. Most of the spinous process and the transverse processes have been broken off. Small foramina occupy the centre of the ventral surface of the centrum. The spinous process appears to have been narrow. Two large foramina penetrate the dorsal surface of the centrum. The caudal extremity of the centrum is circular with a flat articular facet.

The spinous process is craniocaudally narrower relative to the axis than in those of *Z. trilobus.* The dorsal centrum foramina vary in *Z. trilobus* from fused (WAM 65.4.150) to separated (WAM 96.3.402), which may be ontogenetic.

#### 3.2.6. Cervical vertebrae

Three post-axis cervical vertebrae are included in specimen SAMA P54742. All three are highly damaged with only the centrum remaining, making it difficult to determine their order.

#### 3.2.7. Thoracic vertebrae

**Figure 8.**
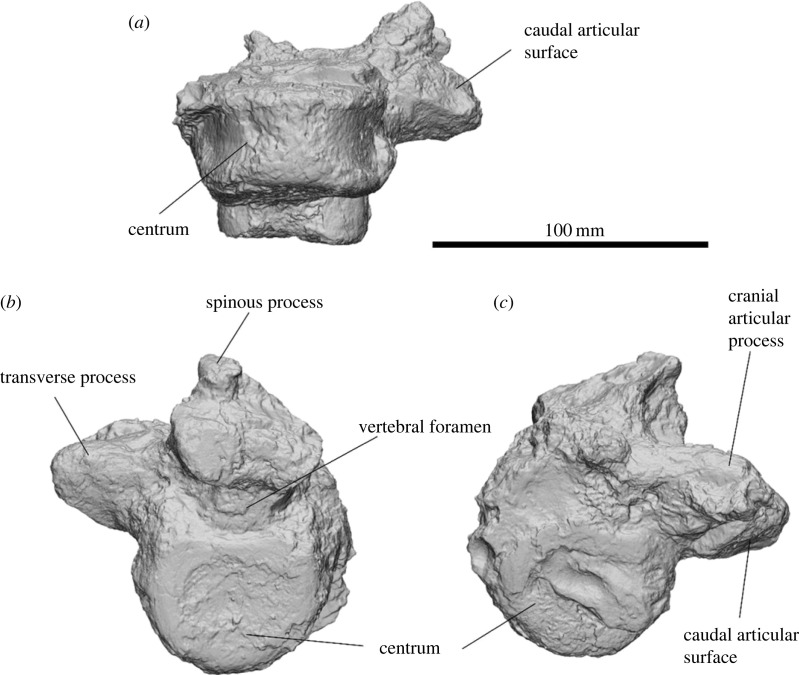
Thoracic vertebra of *Ambulator keanei* (SAMA P54742). Ventral view (*a*), cranial view (*b*) and caudal view (*c*).

Four partial thoracic vertebrae were found with SAMA P54742. Due to their damage, we could not identify them to position, but we have made an attempt to describe them broadly. All possess a large semicircular centrum with flat cranial and caudal extremities (figure 8). A large vertebral foramen is dorsal of the centrum. The transverse processes are broken on both sides, but they appear to have extended laterally and originate level with the vertebral foramen. The caudal articular surfaces for the ribs are angled caudocranially and positioned close to the base of the transverse processes. The cranial articular processes are located at the base of the transverse processes and are angled craniodorsally. The spinous process is broken dorsal to the vertebral foramen.

#### 3.2.8. Clavicle

**Figure 9.**
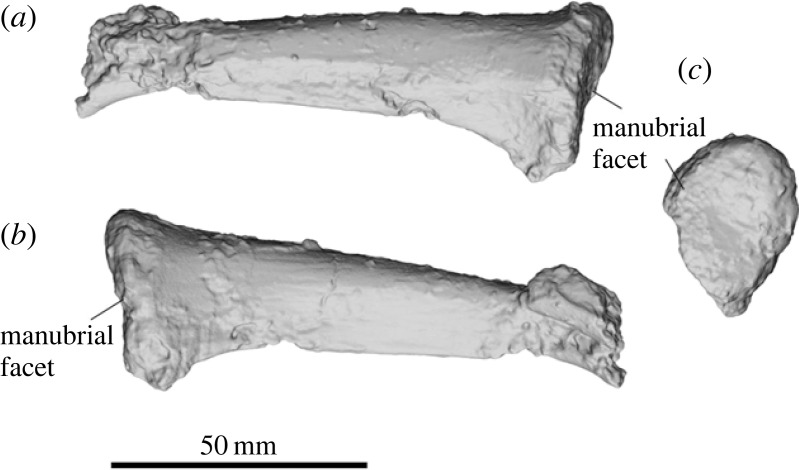
Partial right clavicle of *Ambulator keanei* (SAMA P54742). Cranial view (*a*), caudal view (*b*) and medial view (*c*).

SAMA P54742 preserves one partial clavicle, which is cylindrical and tapers towards the acromial end (figure 9). The shaft is slightly curved, with a strong ridge extending along the entire ventral end of the shaft for attachment of *m. pectoralis major*. The facet for the manubrium is oval, with the articular surface being somewhat concave.

#### 3.2.9. Manubrium and sternebrae

**Figure 10.**
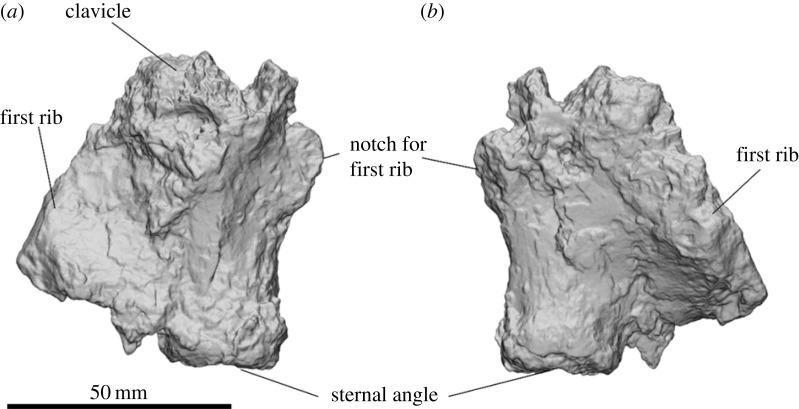
Partial manubrium of *Ambulator keanei* (SAMA P54742). Ventral view (*a*) and dorsal view (*b*).

The manubrium of SAMA P54742 is highly damaged with part of the right clavicle and the first rib crushed into it (figure 10). The cross-section is broad and mediolaterally oval. The cranial end is highly damaged but appears to be broadly crucifix shaped and widest at the cranial edge and narrowest at the caudal. The notch for the first rib projects laterally of the cranial end of the body. The sternal angle is circular with a flat articular surface.

Two sternebrae were found with SAMA P54742, both are rectangular and dorsoventrally compressed.

#### 3.2.10. Ribs

Nine rib fragments are included in SAMA P54742. The first rib is flat and curves medially, and tapers proximally. The sternal ribs are elongated and medially curved, tapering slightly distally; they are flat in section, but are not as broad as the first rib. The articular process of the head is elongated with a spherical articular surface at the proximal end and transitions to posterodistally concave at the distal end. The neck of the ribs is pinched between the head and the tubercle (transverse process facet). The tubercle is located on the medial face of the rib and is small compared to the head. The body varies in thickness and size, but all are curved and taper distally.

#### 3.2.11. Scapula

**Figure 11.**
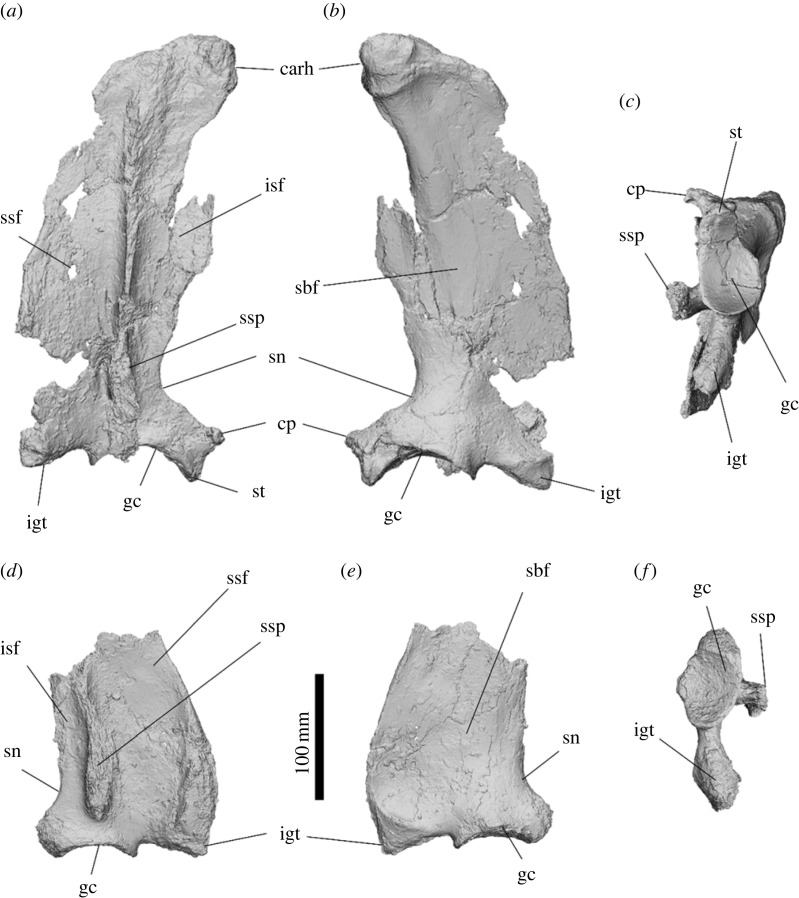
Right scapula (*a*–*c*) and left scapula (*d*–*f*) of *Ambulator keanei* (SAMA P54742). Lateral view (*a*,*d*), medial view (*b*,*e*) and glenoid view (*c*,*f*). Abbreviations: **carh**, cranial angle and insertion for *mm. rhomboids*; **cp**, coracoid process; **gc**, glenoid cavity; **igt**, infraglenoid tubercle; **isf**, infraspinous fossa; **sbf**, subscapular fossa; **sn**, scapular notch; **ssf**, supraspinous fossa; **ssp**, scapular spine; **st**, supraglenoid tubercle/process.

The scapula is triangular when viewed from the lateral aspect, being broadest distally and tapering proximally (figure 11). The scapular spine is abraded and the acromion process is missing in both the left and right scapulae of SAMA P54742; however, it is apparent that both the infraspinatus and supraspinatus fossae are shallow. The scapula is 342.6 mm deep from the glenoid cavity to the cranial angle. The dorsal scapular body curves medially in cranial view. The cranial angle and the insertion for *mm. rhomboids* form a distinct, rounded tip, positioned anterocaudal to the scapular spine. No distinct caudal angle is present, and the dorsal border is rugose with a large insertion scar for *m. serratus posterior* on the proximocaudal face of the spine. The edges of both supraspinous and infraspinous fossae are partially missing, but most of the borders can be inferred. The scapular notch is concave on the medial edge. The oval glenoid cavity is 49 mm × 83 mm and tapers cranially. The glenoid cavity is a concave articular surface, particularly deeply concave when viewed from the lateral aspect*.* The supraglenoid process is a small tubercle on the craniolateral tip of the glenoid fossa. The large laterally projecting coracoid process hooks medially. A large infraglenoid tubercle/process extends 64.5 mm from the glenoid fossa, expanding in width to form a rugose surface for attachment of the *mm. triceps brachii caput longum* and *mm. teres major*.

The scapula is more similar to that of *Z. trilobus* than to *Kolopsis torus.* It differs from those of *Kolopsis torus*, *Pl. centralis* and *Ne. stirtoni* by possessing a large infraglenoid process, a rugose cranial angle projecting slightly cranial of the scapula spine, and a large caudolaterally hooked coracoid process. It differs from that of *Z. trilobus* by being less medially curved around the ribs and possessing a larger hooked coracoid process. It differs from that of *D. optatum* by lacking an infraspinous process on the caudal border of the scapula.

#### 3.2.12. Humerus

The left humerus of SAMA P54742 is highly abraded on the cranial face of the humeral head and the proximal 1/3 of its length. The lateral supracondylar crest and the greater tubercle are abraded off on SAMA P54742. The humerus is relatively gracile for a diprotodontid, with a total length of 324 mm from the distal trochlea to the humeral head, and it has a minimum circumference of 117.6 mm at the distal part of the diaphysis. The humeral head projects slightly caudally. The lesser tubercle is positioned directly below the humeral head, covering the medial and caudomedial sides of the proximal humerus. The deltopectoral crest is relatively straight, extends over more than half of the proximal humerus and terminates level with the deltoid tuberosity just over half-way down the shaft. The maximum height of the deltopectoral crest is on the distal end parallel to the deltoid tuberosity and appears to be an attachment for the *m. deltoideus pars clavicularis*. The deltoid tuberosity for attachment of the *m. deltoideus pars spinalis/acromialis* is on the lateral side, perpendicular to the deltopectoral crest, and tapers gradually both proximally and, more abruptly, distally in a concave slope. The tuberosity for *m. latissimus dorsi* and *m. teres major* projects 1/3 of the way down the medial humeral shaft, forming a small ridge that hooks slightly caudally. A shallow groove for the origin of the *m. brachialis* is situated below the humeral head and wraps below the deltoid tuberosity to meet the cranial face of the lateral supracondylar crest. The olecranon fossa is shallow. The radial fossa is moderately indented above the midline of the trochlea with two very weak convex ridges. The medial and lateral epicondyles extend outward, making the humerus the widest at the distal end. The supracondylar bridge is broken off but the ends show it was 68.45 mm in length. The capitulum is circular on the cranial aspect and projects further cranially than the trochlea. The articular facet of the trochlea is craniocaudally narrower than the capitulum and the lateral aspect is shallowly concave.

**Figure 12.**
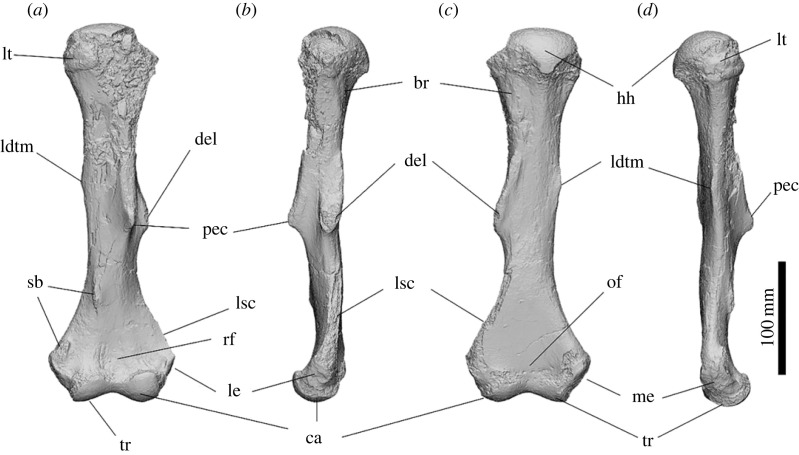
Left humerus of *Ambulator keanei* (SAMA P54742). Cranial view (*a*), lateral view (*b*), caudal view (*c*) and medial view (*d*). Abbreviations: **ca**, capitulum; **del**, deltoid tuberosity; **H**, humeral head; **ldtm**, insertion for *mm. latissimus dorsi* and *teres major*; **le**, lateral epicondyle; **lsc**, lateral supracondylar crest; **lt**, lesser tubercle; **me**, medial epicondyle; **of**, olecranon fossa; **pec**, deltopectoral crest; **rf**, radial fossa; **sf**, supracondylar bridge; **tr**, trochlea.

The overall shape of the humerus is gracile in comparison to those of *Z. trilobus* and *D. optatum*. The caudal projection of the humeral head is similar to those of *Z. trilobus* and is less caudally projected and more ovoid from the proximal aspect than in those of *Ne. stirtoni* and all diprotodontid humeri from Alcoota except NTM P7250. The greater tubercle is damaged and consequently cannot be compared to other diprotodontids. The lesser tubercle is relatively large and positioned more cranially than observed in most diprotodontids but is similar to the condition of diprotodontid humeri from Alcoota. The origin of the *m. brachialis* leaves a rugose lump below the caudolateral side of the humeral head which is similar to, but not as rugose as, that seen in *D. optatum* and *Z. trilobus*. A large insertion scar for *mm. latissimus dorsi* and *teres major* is positioned more proximally than the deltoid tuberosity, similar to that of *Z. trilobus*; this is unlike that of *D. optatum* that has a very weak insertion scar in approximately the same location, and also those of *Ne. stirtoni*, *Ngapakaldia tedfordi*, *Nimbadon lavarackorum* and diprotodontid humeri from Alcoota that possess a large insertion scar level with the deltoid tuberosity. The deltopectoral crest is straight, similar to that seen in *D. optatum*. The deltoid tuberosity is large, similar to those of *D. optatum* and *Z. trilobus*. The supracondylar bridge is present, differentiating it from that of *D. optatum* in which the supracondylar bridge was cartilaginous or ligamentous. The full extent of the lateral supracondylar crest cannot be determined, but it appears to be better developed than that observed in *D. optatum.* The medial epicondyle is relatively large, similar to those of *D. optatum*, *Z. trilobus* and *Kolopsis torus*, providing a greater attachment area for the *anconeus medialis*, *m. palmaris longus*, *m. flexor carpi radialis*, *m. flexor carpi ulnaris* and *m. flexor digitorum profundus.* The trochlea is rounded and angled slightly cranially, similar to in *D. optatum*, as opposed to the flatter trochlea observed in *Ngapakaldia* (UCMP 69814 and UCMP 75968), or the rounded but less spherical trochlea in that of *Z. trilobus* (figure 12).

#### 3.2.13. Ulna

**Figure 13.**
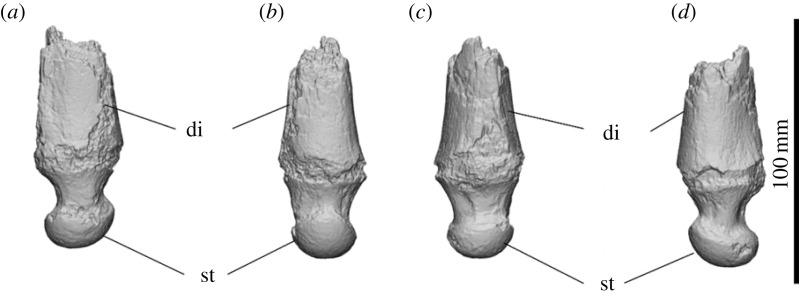
Distal left ulna of *Ambulator keanei* (SAMA P54742). Lateral view (*a*), caudal view (*b*), cranial view (*c*) and medial view (*d*). Abbreviations: **di**, diaphysis; **st**, styloid process.

Distal pieces of the left ulna with the styloid process are known from SAMA P54742 and SAMA P52377. Abrasion is present all around the epiphyseal junction. The base of the diaphysis widens at the epiphyseal junction. A more convex edge is present on the cranial face of the diaphysis. The styloid process is slightly caudally deflected, tapering from the diaphysis to a large bulb with a distinct convex articular surface for the triquetrum and pisiform (figure 13). The styloid process is egg shaped in distal view and smaller in diameter than the distal diaphysis; this bulb is widest at the cranial edge and tapers to a rounded point on the caudal face. No visible grooves for the *m. extensor carpi radialis* and *m. extensor carpi minimi*.

The styloid process of the ulna is most similar to those of *Z. trilobus* and *D. optatum*, being large and proximally deflected, but less so than in *D. optatum.* The oval styloid process is more similar to that of c.f. *Eur. dunense* (VH121) than it is to the more spherical styloid in those of *Z. trilobus* or *D. optatum.* The styloid process of *Am. keanei* differs from those of all Alcoota diprotodontids by being proportionally larger in comparison to the diameter of the shaft.

#### 3.2.14. Radius

**Figure 14.**
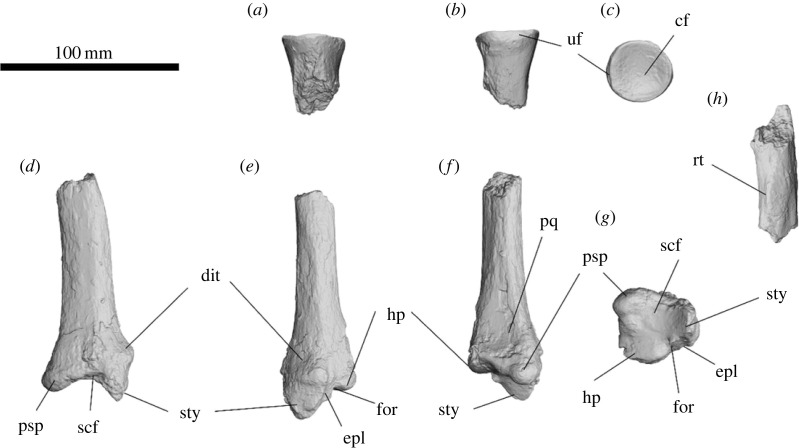
Partial left radius of *Ambulator keanei* (SAMA P54742) (*a–g*) and a proximal radial diaphysis of undetermined side (*h*). Proximal fragment (*a–c*), distal fragments (*d–g*), lateral view (*a*,*e*), caudal view (*b*,*f*), proximal view (*c*), medial view (*d*) and distal view (*g*). Abbreviations: **cf**, capitular facet; **dit**, distal tuberosity; **for**, foramen; **epl**, groove for *m. extensor pollicis longus*; **hp**, hamatum process; notch for *m. pronator quadratus*; **psp**, posterior scaphoid process; **pq**, notch for the *m. pronator quadratus*; **rt**, radial tuberosity; **scf**, scaphoidal facet; **sty**, styloid process; **uf**, ulnar facet.

The capitular facet is circular and deeply cupped (figure 14). The ulnar facet is a thin strip extending across the medial border of the capitular facet. The diaphysis is incomplete but appears to first taper from the capitular facet only to widen again towards the distal end and is triangular in cross-section. The radial tuberosity is distal to the ulnar facet forming a weak rugose protuberance. The notch for the *m. pronator quadratus* is triangular and deeply concave, on the medial face of the shaft widening distally before terminating at the distal epiphysis. The carpal surface is sub-triangular when viewed from the distal aspect, with the scaphoid facet extending between the styloid process and the posterior scaphoid process. The scaphoid facet is oval and anteroposteriorly elongate from the distal aspect and deeply concave from lateral aspect. The styloid process tapers to a rounded point in lateral aspect and is anteroposteriorly compressed, forming a sharp point in caudal aspect. A large, rugose distal radial tuberosity is positioned above the styloid process, at the epiphyseal border. The posterior scaphoid process is rounded to a bulb that is slightly deflected medially. The hamatum process extends medially from the scaphoid facet towards the hamatum; the surface is relatively flat compared to the scaphoid facet and forms a wide rounded medial edge. A small barrel-shaped concavity lies on the medial face between the styloid and hamatum facet, possibly for insertion of a ligament. The groove for the *m. extensor pollicis longus* is on the medial face of the styloid facet and is very shallow.

The ulnar facet is similar in size and shape to that of *Z. trilobus* and smaller than in those of all diprotodontids from Alcoota. The notch for the *m. pronator quadratus* is more similar to that of *Z. trilobus* and smaller than in some diprotodontid radii from Alcoota, such as NTM P7319 and NTM P6141, but not others, such as NTM MP1328. The diaphysis cross-section is similar in shape to that of *Z. trilobus*. The distal articular surface of the radius is also more similar to that of *Z. trilobus* than to any other diprotodontid observed in this study. The hamatum process is proportionally larger than in most diprotodontid radii from Alcoota, especially NTM P7325 and NTM P6141, but is not that much larger than in NTM MP1328. The extensive hamatum process results in a triangular appearance on the distal aspect of the shaft, similar to that of *Z. trilobus*. The size and shape of the scaphoid facet, anterior scaphoid process, and the styloid process, appear to be within the range of variation of *Z. trilobus* TMAGZ 3554 and WAM 68.1.29.

#### 3.2.15. Manus

**Figure 15.**
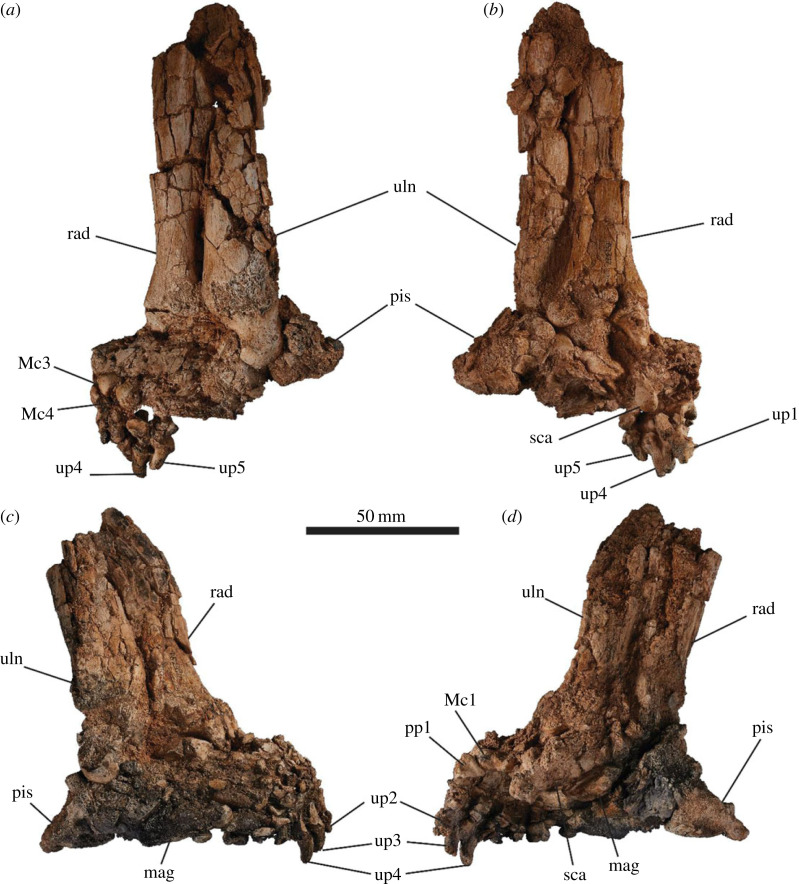
Articulated left (*a*,*b*) and right (*c*,*d*) manus of SAMA P52377. Medial view (*b*,*d*) and lateral view (*a*,*c*). Abbreviations: **mag**, magnum; **Mc**, metacarpal; **pp**, proximal phalanx; **pis**, pisiform; **rad**, radius; **sca**, scaphoid; **uln**, ulna; **up**, ungual phalanx.

The carpals and metacarpals are very large relative to the digits (figure 15). The pisiform is orientated semi-horizontally so that its plantar surface forms a heel that is weight bearing. The metacarpo-phalangeal junction is highly damaged and distorted in SAMA P52377. The articular facets between the proximal and medial phalanges in SAMA P54742 result in the phalanges being angled dorsally above the rest of the plantar surface of the manus in a slightly crimped position.

**Figure 16.**
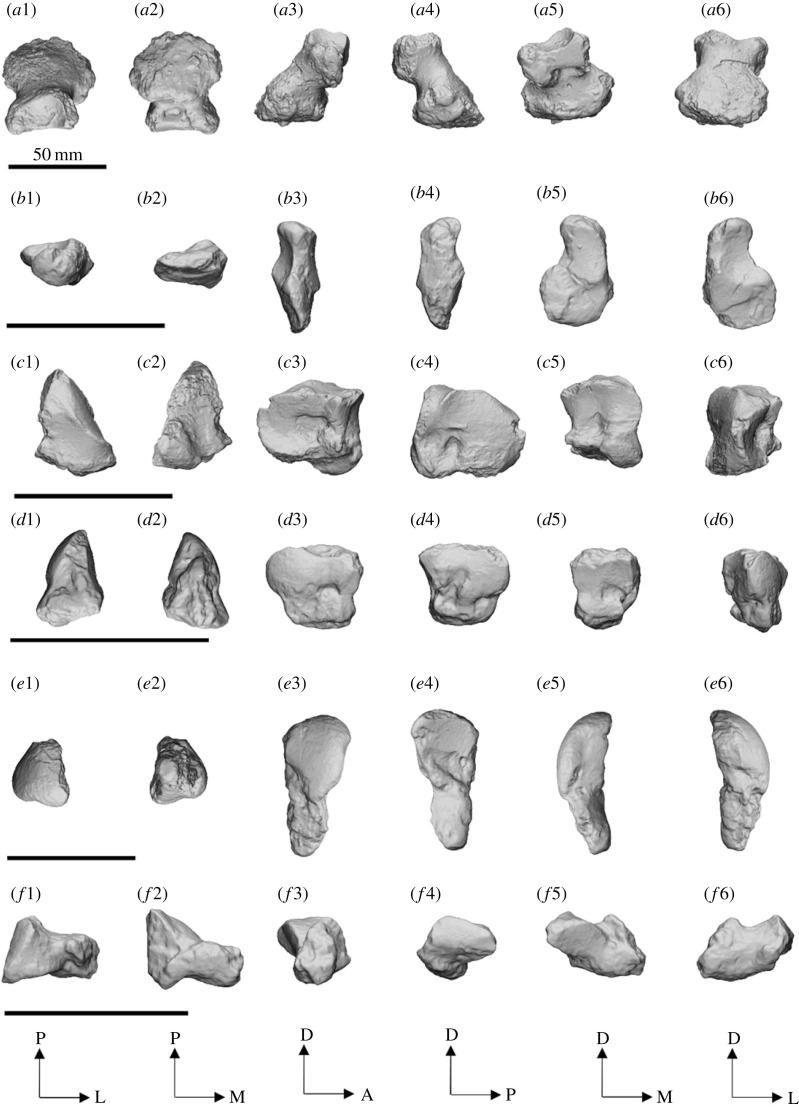
Left carpals of *Ambulator keanei* (SAMA P54742). Pisiform (*a*), triquetrum (*b*), hamatum (*c*), capitatum (*d*), scaphoid (*e*) and right trapezium (*f*). Carpals were difficult to orientate but broadly the views presented here are dorsal view (1), plantar view (2), medial view (3), lateral view (4), cranial (anterior) view (5), caudal (posterior) view (6).

The pisiform (figure 16*a*) is hourglass shaped in dorsal view, with a rugose, caudally projecting tuber for attachment of the *m. flexor carpi ulnaris* and a concave cranial surface. It is anteroposteriorly elongated, widest at the tuber, pinches to a thin waist at the centre and widens again at the stylar facet. The tuber is wide and is positioned at an approximately 45° angle relative to the ground; it also has a large, flat, palmar component. The stylar facet is large and cup shaped, covering most of the cranial face; it is angled craniodorsally, supports the majority of the styloid process of the ulna, is widest on the lateral side and tapers medially. Two triquetral facets are present on the cranial face: one is small and triangular, confined to the craniomedial face, palmar to the styloid facet; the other is a small and oval, on the craniolateral face. The triquetral fossa is large, deep and oval, positioned on the antero-palmar face, caudal to the triquetral facet. The pisiform of SAMA P52377 (figure 15) shows contact with the proximal scaphoid facet of the radius dorsal to the hamatal facet of the pisiform but not enough to distinguish a facet.

The expanded distal end provides a large attachment area for the *m. flexor carpi ulnaris* and is more similar to that seen in diprotodontid pisiforms from Alcoota and those of *D. optatum* than that of *Z. trilobus* (figure 18)*.* The elongated waist differentiates *Am. keanei* from *D. optatum*. The larger styloid fossa and small, dorsally angled triquetral fossa are more similar to those in *Z. trilobus* and *D. optatum* than in diprotodontid pisiforms from Alcoota. The large ligamental fossa is similar to that in NTMP 4334 but is unlike that in other diprotodontid pisiforms from Alcoota or those of *Z. trilobus*.

The triquetrum (figure 16*b*) is small compared to most other carpals; it is thin and dorsoplantarly elongated. The pisiform facet is small and triangular on the posterior face. The styloid facet is large (approx. 2/3 the size of the pisiform stylar facet), crescent shaped and cups the anteroplantar surface of the styloid process, being largely concave on the medial side. The hamatal facet is dorsoplantarly elongated and split into two parts covering most of the anterior face; the dorsal facet is larger and shallowly saddle shaped and the palmar facet is smaller and flat.

The triquetrum differs from those of all other diprotodontids by being smaller in proportion to the rest of the manus. It has a narrower hamatal facet that is angled at approximately 45° to the styloid facet, as opposed to nearly 90° as observed in that of *Z. trilobus* (WAM 2023.4.14). The crescent-shaped styloid facet is similar to that seen in NTM P7268 from Alcoota.

The hamatum (figure 16*c*) is triangular in dorsal view with a rugose palmar face. The triquetral facet is large and bean shaped, covering most of the caudolateral face; the dorsal surface of the facet is slightly saddle shaped, which restricts the mediolateral slippage of the triquetrum. The scaphoid facet continues from the dorsomedial border of the triquetral facet and slopes anteriorly along the dorsal surface of the hamatum. The capitatal facet covers the entire medial face and is deeply concave to lock and restrict the movement of the capitatum. The capitatal fossa is large and positioned in the centre of the capitatal facet. The hamate process is restricted to the cranial end of the palmomedial face, is short, wide and bulbous and does not project cranially below the metacarpal 5 facet. The entire dorsal half of the anterior face is composed of facets for metacarpal 4 and metacarpal 5; the metacarpal 5 facet is the larger of the two, with both facets having a saddle-shaped surface with the border between them being comparatively concave. A shallow fossa is located palmar of both facets.

The hamatum–capitatum ligamental fossa is larger than in that of *Z. trilobus* and *Ne. stirtoni.* The triquetral facet is relatively smaller in proportion to the hamatum than in that of *Z. trilobus* and more similar in shape to those of diprotodontid hamata from Alcoota. A scaphoid facet is present unlike in those of *Z. trilobus* and NTM P7272 from Alcoota and is similar to those of *D. optatum* and NTM P4469 from Alcoota but much smaller relative to the hamatum than in *D. optatum*. The hamate process is larger and more plantarly projected than in those of *Z. trilobus* and *D. optatum*, but smaller than those of *Ni. stirtoni* and diprotodontid hamata from Alcoota (figure 18)*.* The capitatal facet is more concave than NTM P4037 and *Z. trilobus* (SAMA P58502), restricting movement between the hamatum and the capitatum, similar to diprotodontid hamata NTM P4469 and NTM P4469 from Alcoota. The stepped separation of the metacarpal 4 and metacarpal 5 facets that are restricted to the dorsal half of the distal end is more similar to the state in Alcoota diprotodontids than in that of *Z. trilobus*.

The capitatum (figure 16*d*) is triangular in dorsal and ventral aspects, and it tapers plantarly to a rugose face. The hamatal facet is located on the lateral face and mirrors the capitatal facet on the hamatum, which dramatically restricts movement between the two carpals; fossae are located anteroplantar and posteroplantar of the hamatal facet. The scaphoid facet takes up the caudodorsal third of the medial face; it is convex, oval and anteroposteriorly elongated. The trapezoidal facet begins at the anterior border of the scaphoid facet and is the smallest facet on the medial face of the capitatum; its surface is distinctly concave, to contrast the convex surface of the scaphoid facet, and it terminates at the posterior border of the metacarpal 2 facet. The scaphoid fossa is located on the medial face palmar to the trapezoidal facet and posterior of the metacarpal 2 facet. The metacarpal 2 facet takes up the entire anterior border of the medial face; its surface is concave and pinched in the centre. The anterior face is composed of facets for metacarpal 3 and metacarpal 4. The metacarpal 3 facet takes up the majority of the anterior face with a distinct mediolaterally convex surface. The metacarpal 4 facet is comparatively flat and only occupies the dorsolateral corner of the anterior face. A shallow fossa is located ventral to the metacarpal 4 facet and lateral to the metacarpal 3 facet.

The capitatum of *Am. keanei* is craniocaudally shorter than that of *Z. trilobus*. The capitatum is mediolaterally broader than those of diprotodontid capitata NTM P4011 and NTM P4034 from Alcoota. The hamatal and scaphoidal facets converge to a ridge, unlike in that of *Z. trilobus* (WAM 2023.4.18). The hamatal and scaphoidal facets are smaller than in that of *Z. trilobus* and only cover the dorsal halves of both faces. It greatly differs from the state in that of *Z. trilobus* by lacking a plantar extension/process in the metacarpal 3 facet, and in the lack of a ligamental fossa on the metacarpal 4 facet. The facet for metacarpal 2 is much larger than in that of *Z. trilobus* (WAM 2023.4.18).

The scaphoid (figure 16*e*) is long, is widest proximally and tapers palmomedially to a rounded bulb. It articulates with the capitatum, trapezium and the trapezoid. The capitatal facet is located palmolaterally, extending along the entire lateral edge of the scaphoid; it is oval with a concave surface. The capitatal fossa is located distal of the capitatal facet, midway along the lateral face, extending the entire length of the lateral face. The radial facet is a highly convex surface, forming a ball and socket joint with the scaphoid facet of the radius; it occupies entire dorsal surface. The trapezoidal facet is cranially oriented and bean shaped and flat. The trapezium facet is flat and covers the mediodorsal face of the palmar process.

The radial facet of the scaphoid of both *Am. keanei* and those of *Z. trilobus* is concave, unlike the convex facets seen in diprotodontid scaphoids from Alcoota. The trapezoidal facet is noticeably smaller than in that of *Z. trilobus* and is approximately equal in size/slightly larger than in those of all diprotodontid scaphoids from Alcoota. It lacks the large, differentiated hamatal facet that is observed in that of *D. optatum*.

The trapezoid is crushed in both manus of SAMA P52377 and cannot be described.

A partial right trapezium (figure 16*f*) is present in each of SAMA P54742 and SAMA P52377. It articulates with the scaphoid, trapezoid, metacarpal 1 and metacarpal 2. It is roughly hourglass shaped in plantar view and widest at the trapezoid articular border. The trapezoidal border is triangular, narrowing dorsally, with a flat articular surface and is located on the lateral face. The metacarpal 2 facet is small and triangular, located on the dorsomedial corner at the convergence trapezoidal facet and metacarpal 1 facet. The metacarpal 1 facet takes up the entire medial face, has a mediolaterally concave articular surface and is hourglass shaped. The scaphoidal facet is located on the lateral face; it is circular in shape with a weakly convex articular surface.

No trapezium for *Z. trilobus* is known for comparison. That of *Am. keanei* differs from NTM P6730 from Alcoota in having a small scaphoid facet and the metacarpal 1 trapezoidal facets converging to a sharp triangular point.

Metacarpal 1 is the broadest and shortest of the five metacarpals in SAMAP 52377. It is noticeably rectangular in dorsal aspect and is craniodorsally flattened. It articulates with the trapezoid, metacarpal 2 and proximal phalanx 1. The proximal articular surface is broad and dorsoplantarly concave.

Metacarpals 2 to 5 are more elongated than metacarpal 1, and the distal articular surfaces end in a spherical phalangeal facets.

**Figure 17.**
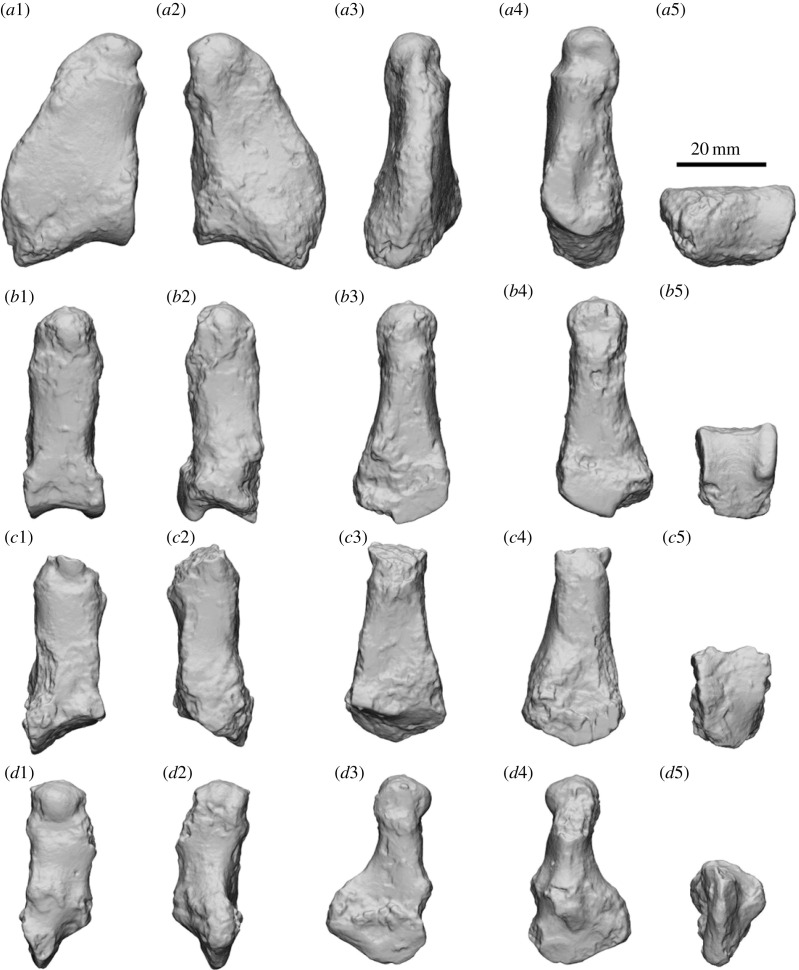
Left metacarpals of *Ambulator keanei* (SAMA P54742). Metacarpal 5 (*a*), metacarpal 4 (*b*), metacarpal 3 (*c*) and metacarpal 2 (*d*). Dorsal view (1), plantar view (2), medial view (3), lateral view (4) and proximal view (5).

Metacarpal 2 (figure 17*d*) is the second shortest metacarpal and articulates with the trapezoid, trapezium, capitatum, metacarpal 3 and proximal phalanx 2. The metacarpal 3, capitatal and trapezoidal facets converge to form a pointed proximal articular surface. The capitatal facet is separated into two parts (upper and lower), with an articular surface mirroring the metacarpal 2 facet on the capitatum with little movement between the two. The metacarpal 3 facet is small and indistinguishable from the dorsal section of the capitatal facet. The trapezoidal facet is small, circular and located dorsomedially on the proximal end. The diaphysis is kinked laterally, triangular in cross-section, and arched in the lateral aspect.

Metacarpal 3 (figure 17*c*) is straight, elongate and cylindrical. It articulates with the metacarpal 2, metacarpal 4, the capitatum and the proximal phalanx 3. The capitatal facet is the largest, taking up all of the proximal articular surface; it is a palmarly tapering trapezoid that is slightly concave and angled caudomedially so that it is convergent on a sharp edge with the metacarpal 4 facet. The metacarpal 2 and metacarpal 4 facets are on opposing edges of the capitatal facet, facing medially and laterally, respectively; both have flat articular surfaces, with that for metacarpal 4 being larger than that for metacarpal 2. The diaphysis is roughly square in cross-section and elongate, resembling a rectangular prism.

Metacarpal 4 (figure 17*b*) is straight, elongate and cylindrical. It articulates with the metacarpal 3, metacarpal 5, capitatum, hamatum and proximal phalanx 4. The hamatal facet is large, taking up most of the proximal articular surface; it is deeply concave, forming a valley between the dorsal and plantar edges. The capitatal facet is small and slightly convex; it extends down the medial edge of the hamatal facet/proximal edge of the metacarpal 3 facet. The facets for metacarpal 3 and metacarpal 5 are on opposing edges of the proximal articular surface, facing medially and laterally respectively; both are flat, with the facet for metacarpal 3 being broader than that for metacarpal 5, but unlike in metacarpal 5, they are restricted to the dorsal half of metacarpal 4. The diaphysis is cylindrical and elongate.

Metacarpal 5 (figure 17*a*) is the largest metacarpal and articulates with the hamatum, metacarpal 4 and proximal phalanx 5; it is triangular with a slightly convex lateral edge in dorsal aspect. The hamatal facet takes up most of the proximal articular surface; the articular surface is palmodorsally convex and mediolaterally concave. The metacarpal 4 facet is on the medial edge of the hamatal facet and forms an articular surface that is flat and faces medially. The diaphysis is broad and plantar-dorsally flattened, the medial edge is flat, while the lateral edge is convex, and it tapers distally.

**Figure 18.**
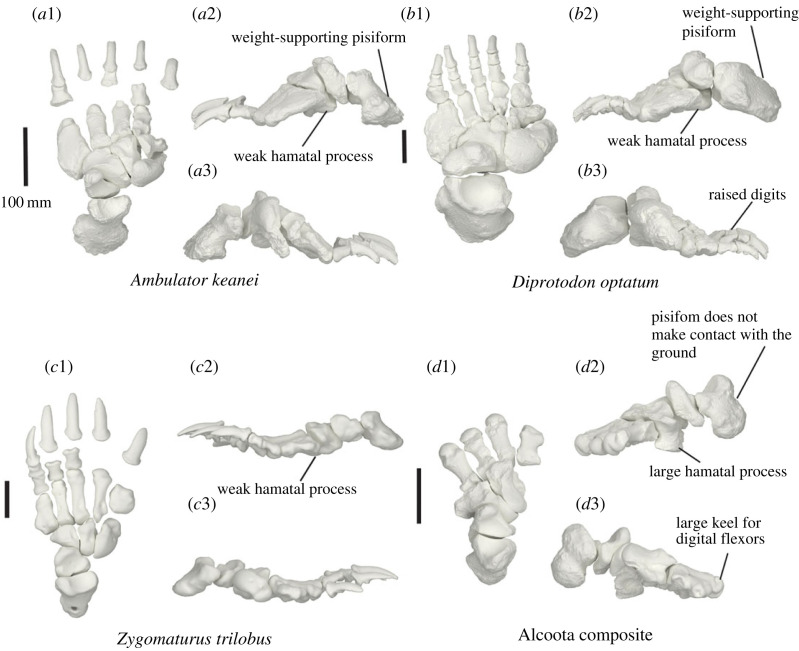
Left manus of *Ambulator keanei* (SAMA P54742) (*a*), right manus (mirrored) of *Diprotodon optatum* (SAMA P5120) (*b*), right manus (mirrored) of *Zygomaturus trilobus* (TMAG Z3554) (*c*), composite right manus (mirrored) of an Alcoota diprotodontid (*d*). Dorsal view (1), medial view (2) and lateral view (3). Digits positioned at maximum extension at the metacarpal–phalangeal joint.

The metacarpals are notably shorter, and taper anteriorly more, than in those of *Z. trilobus*. They differ from those of all diprotodontid metacarpals from Alcoota by lacking a keel for separation of the sesamoids on all distal articular facets (figure 18). Metacarpal 5 in *Am. keanei*, unlike in those of *Z. trilobus*, is the largest metacarpal and is triangular in dorsal view with a convex lateral edge, as in that of *D. optatum* (figure 18).

All proximal and middle phalanges are too damaged and fragmented in SAMA P52377 to provide useful information; only the proximal phalanx 2 and 3 and middle phalanges 2 are present in SAMA P54742.

The proximal phalanges are longer than middle phalanges in all digits and are hourglass shaped with a semicircular cross-section. The proximal articular surface is slightly concave; it extends further proximally on the medial border. A tuberosity is present on the lateral side of the proximal articular facet. The distal articular facet is slightly concave and positioned on the distal plantar surface, limiting movement between the proximal phalanx and middle phalanx in all digits, and is angled slightly medially in SAMA P52377.

**Figure 19.**
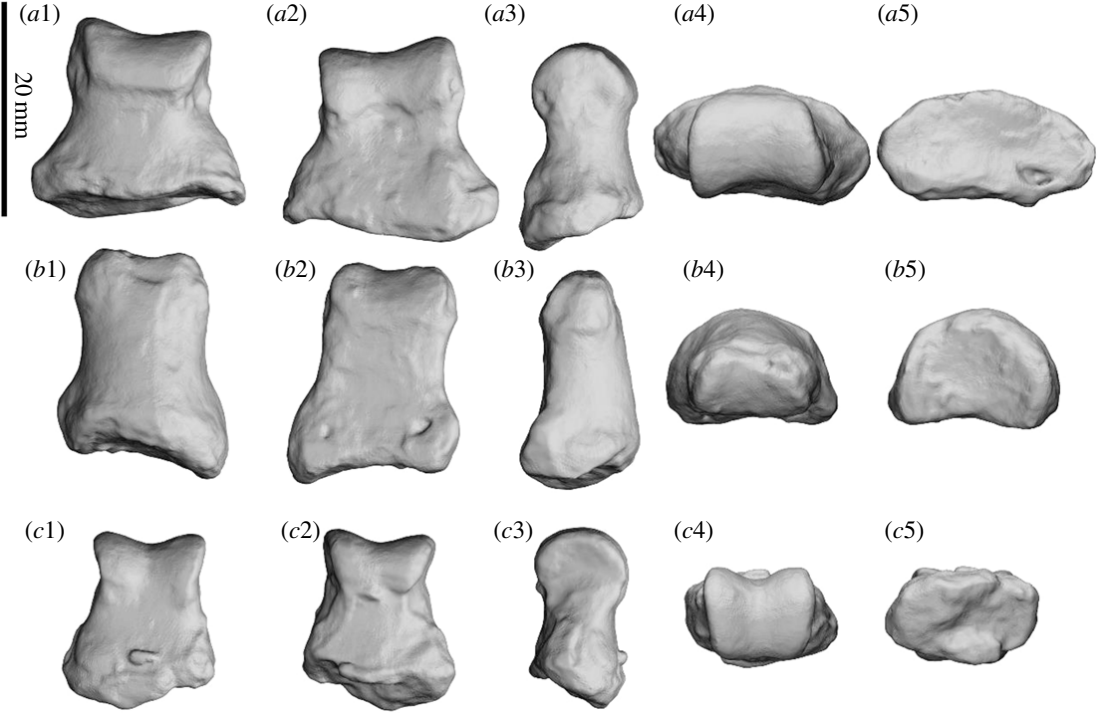
Left manual phalanges of *Ambulator keanei* (SAMA P54742). Middle phalanx 5 (*a*), proximal phalanx 2 (*b*) and middle phalanx 3 (*c*). Dorsal view (1), plantar view (2), lateral view (3), proximal view (4) and distal view (5).

The middle phalanx 5 (figure 19*a*) is broader than all other phalanges, with a wider proximal articular facet and shallow median keel; it is hourglass shaped and oval in cross-section. The distal articular surfaces are saddle shaped; this indicates more dorsoventral movement occurs between the ungual and middle phalanx than between the middle phalanx and proximal phalanx.

**Figure 20.**
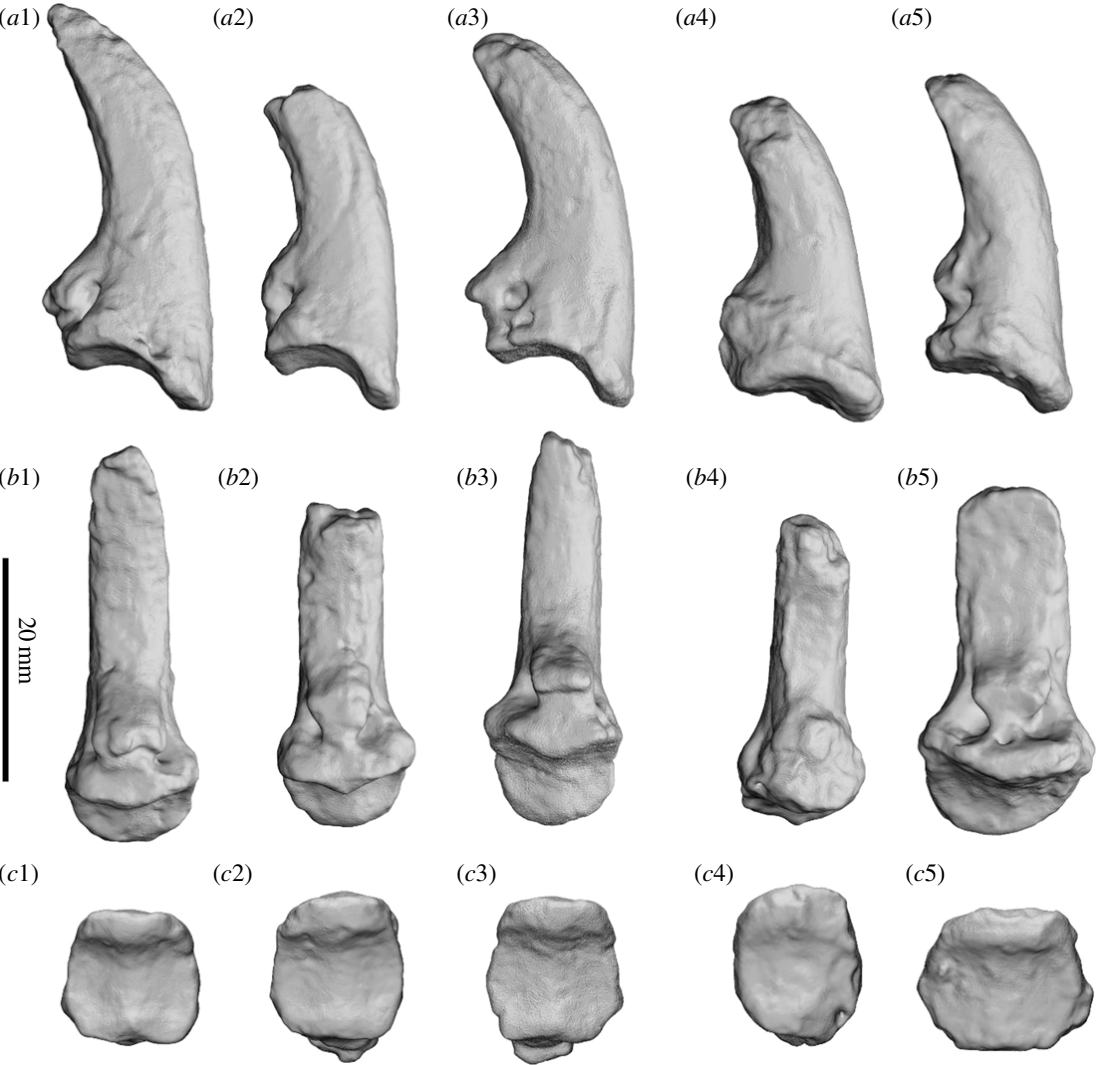
Left ungual phalanges of *Ambulator keanei* (SAMA P54742). Ungual phalanx 5 (1), ungual phalanx 4 (2), ungual phalanx 3 (3), ungual phalanx 2 (4) and ungual phalanx 1 (5). Lateral view (*a*), plantar view (*b*) and proximal view (*c*).

The ungual phalanges (figures 20) are relatively long compared to the proximal and middle phalanges, with all but ungual phalanx 1 being dorsoplantarly taller than wide. The proximal articular facets are deeply dorsopalmarly concave while being slightly mediolaterally convex. All phalanges have large arch-shaped articular facets with a faint median keel present. All ungual phalanges are elongated and narrow except ungual phalanx 1. Ungual phalanx 1 is wider and flatter than all other unguals. The distal tip is missing for most unguals, but ungual phalanx 3 appears to be the longest. The flexor tubercles are narrower than the articular facets; a deep fossa is present on the lateral and the medial sides for attachment of the flexor tendons.

The proximal and middle phalanges are relatively shorter and taper more anteriorly than in those of *Z. trilobus*, similar to that seen in *D. optatum*. The ungual phalanges of *Am. keanei* are narrower and taller relative to length than in those of *Z. trilobus* and do not display any of the characteristic dorsopalmar flattening seen in the latter with the exception of ungual phalanx 1.

#### 3.2.16. Femur

**Figure 21.**
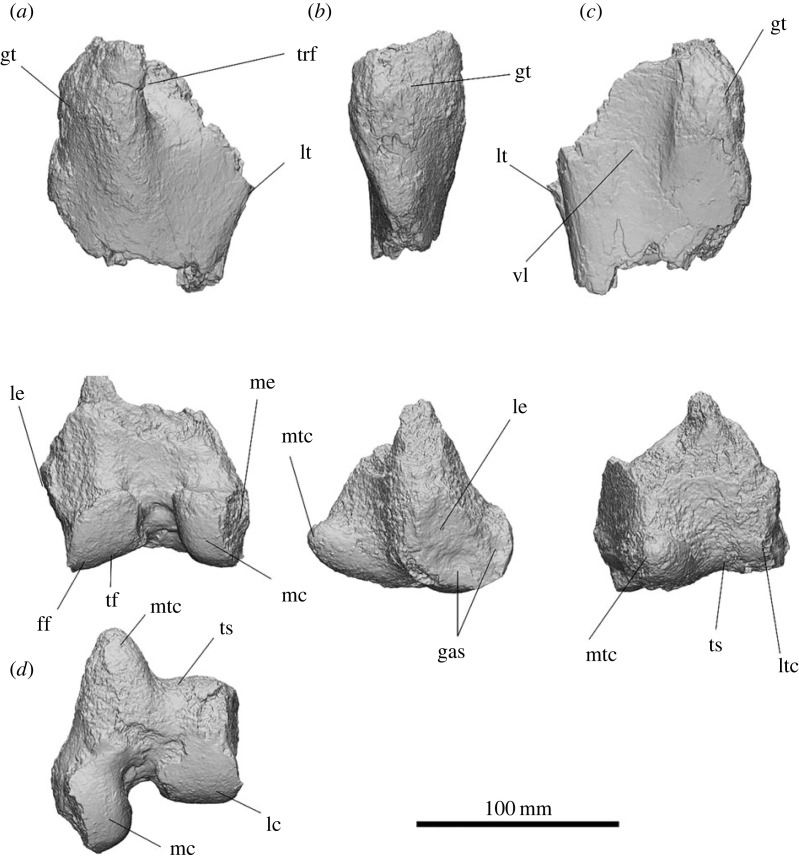
Partial left femur of *Ambulator keanei* (SAMA P54742). Caudal view (*a*), medial view (*b*), cranial view (*c*) and distal view (*d*). Abbreviations: **fab**, fabella facet; **ff**, fibular facet; **gas**, origin for *m. gastrocnemius*; **gt**, greater trochanter; **lc**, lateral condyle; **le**, lateral epicondyle; **ltc**, lateral trochlear crest; **lt**, lesser trochanter; **mc**, medial condyle; **me**, medial epicondyle; **mtc**, medial trochlear crest; **tf**, tibial facet; **trf**, trochanteric fossa; **ts**, trochlear surface; **vl**, origin for *m. vastus lateralis*.

A partial left femur of SAMA P54742 (figure 21) is broken into two parts: a proximal fragment, preserving part of the greater trochanter and shaft, and the near-complete distal end. At the proximal end, the diaphysis is thickest at the greater trochanter on the lateral side, and width tapers substantially towards the medial face. The greater trochanter tapers distally to merge with the diaphysis and its surface is rugose. The surface of the cranial face of the diaphysis is smooth and mediolaterally concave creating a shallow sulcus for part of the origin of the *m. vastus lateralis.* The proximal half of the trochanteric fossa is partially missing, but the remnant is broad and faces medially.

The distal femoral fragment is broken at the diaphysis, with the edges of most features being abraded to some extent. The attachment for the *m. gastrocnemius medialis* is separated into two indentations on the border of the lateral epicondyle. The lateral condyle is broader than the medial condyle and aligned in the sagittal plane; it possesses a flatter articular surface, for articulation with the tibia, than the more rounded medial condyle; a small articular facet for the fibula is on the lateral border. The medial condyle is abraded on the medial face, but its full width still appears substantially narrower than the lateral condyle. When viewed from the distal aspect, the medial condyle extends far more caudally than the lateral condyle and is rotated laterally out of the sagittal plane; the articular surface is more rounded. The trochlea surface is deeply concave on the cranial face. The medial trochlea crest is cone shaped and protrudes substantially cranially in comparison to the rest of the trochlea. The lateral surface of the trochlea is partially abraded but does not appear to extend to the same extent as the medial trochlear crest.

The femur of *Am. keanei* is most similar to that of *D. optatum* and differs by lacking the slight step in the lateral condyle seen in *D. optatum*. The large medial trochlear crest is most similar in shape to that in *D. optatum*. That *Am. keanei* lacks a large distinct step in the lateral condyle for separation of the tibial and fibular facets is most similar to that in some diprotodontid femora from Alcoota, such as NTM P4130, in contrast with those of *Z. trilobus*. The femur of *Am. keanei* differs from that of *Z. trilobus* in the absence of the lateral step for the fibula on the lateral condyle and a weaker medial crest. The lateral crest is broken in all specimens of *Z. trilobus*, but the base is proportionally larger in *Am. keanei*. The trochlear surface extends less cranioproximally than in *Z. trilobus*.

#### 3.2.17. Tibia

**Figure 22.**
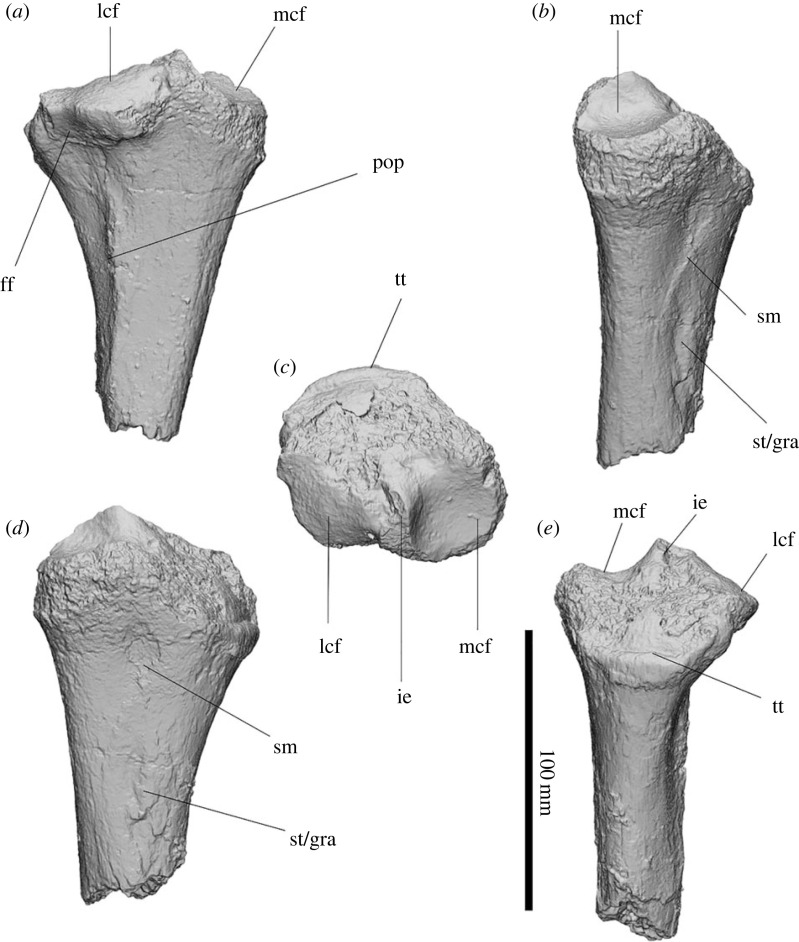
Partial left tibia of *Ambulator keanei* (SAMA P54742). Caudal view (*a*), medial view (*b*), proximal view (*c*), cranial view (*d*), lateral view (*e*). Abbreviations: **ff**, fibular facet; **gra**, insertion scar for *m. gracilis*; **ie**, intercondylar eminence; **lcf**, lateral condyle facet; **mcf**, medial condyle facet; **pop**, popliteal fossa; **sm**, insertion scar for *m. semimembranosus*; **st**, insertion scar for *m. semitendinosus*; **tt**, tibial tuberosity.

Only the proximal end of the tibia is known (SAMA P54742) (figure 22). It is broadest at the femoral facet and gradually tapers distally down the diaphysis. The medial condyle facet is circular, its surface is deeply concave and it has a distinct border next to the attachment scar for the ligamentum trochleae. The lateral femoral facet is triangular in dorsal view and is angled caudally relative to the tibial head. The articular surface is slightly convex over the medial half but is slightly concave on the lateral edge near the fibular facet. The fibular facet is roughly rectangular in shape and shares a border with the lateral femoral facet; the cranial half is slightly craniocaudally concave before becoming slightly convex towards the caudal half, creating a tighter fit for the fibula. A large intercondylar eminence separates the lateral and medial femoral facets. The tibial tuberosity is sloped craniodistally and occupies approximately half of the proximal face. The insertion scar for *m. popliteus* forms a large, slightly curved ridge travelling down the caudal face below the fibula facet. The insertion scar of *m. semimembranosus* forms a small indentation on the craniomedial face just below the border of the medial condyle facet and the tibial tuberosity. The insertion scars for the *m. semitendinosus* and *m. gracilis* are faint and share an indentation distal of the insertion scar for the *m. semimembranosus* 1/3 further down the shaft.

The proximal tibia of *Am. keanei* is morphologically very similar to that of *Z. trilobus*. The tibial tuberosity is less laterally extensive than in that of *Z. trilobus*. The surface of the lateral femoral facet is angled more dorsally relative to the medial femoral facet similar to those of *D. optatum* and *Euo. grata* and unlike the more caudally angled lateral femoral facet in that of *Z. trilobus*. No medial projection of the medial femoral facet like in that of *D. optatum* is observed in *Am. keanei*. The attachment for *m. popliteus* is positioned slightly more laterally on the diaphysis below the apex of the fibula/lateral femoral condyle facet than in that of *Z. trilobus*.

#### 3.2.18. Fibula

**Figure 23.**
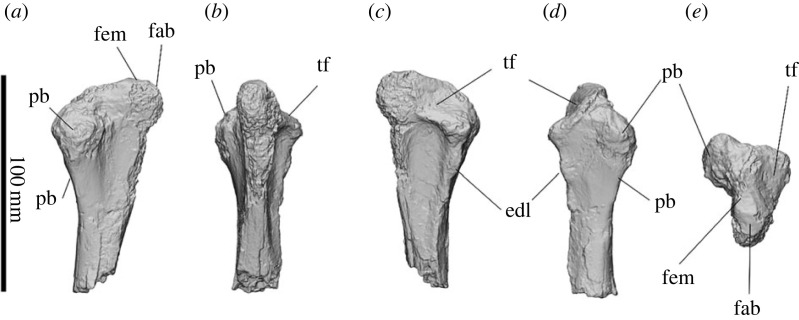
Partial left fibula of *Ambulator keanei* (SAMA P54742). Lateral view (*a*), caudal view (*b*), medial view (*c*), cranial view (*d*), proximal view (*e*). Abbreviations: **edl**, attachment for *m. extensor digitorum longus*; **fab**, fabella facet; **fem**, femoral facet; **pb**, attachment for *m. peroneus brevis*; **tf**, tibial fossa.

Only the proximal portion of the fibula is known (SAMA P54742) (figure 23); it is triangular in cross-section, pinching caudally; it is craniocaudally 54.9 mm deep and mediolaterally 41.6 mm wide; it tapers distally from the fibular head to the diaphysis. The tibial facet is the largest and occupies almost half of the dorsomedial surface; the surface is slightly concave. The femoral facet is small (one-tenth the size of the tibial facet) and triangular, located on the medial side of the tibial facet, and shares a raised border with the fabellar facet. The fabellar facet is partially abraded but appears to be slightly larger than the femoral facet and is positioned caudal to it. The attachment scar for *m. peroneus brevis* is large and rugose, taking up the entire craniolateral corner of the fibular head. The ridge for the attachment of *m. extensor digitorum longus* is below the cranial corner of the fibular facet and is rugose.

The comparatively tiny femoral facet of the fibula of *Am. keanei* is in stark contrast to the large concave one in that of *Z. trilobus*. The femoral facet is smaller than any other diprotodontid examined in this study, and only those of *Ni. lavarackorum*, *Ng. tedfordi* and some diprotodontid femora from Alcoota such as NTM P92756 and NTM P4827 are close to it in such small size relative to the rest of the fibular head. The relatively larger tibial facet in *Am. keanei*, covering half of the fibula head and with a more laterally extended process for attachment of *m. peroneus digiti quartus*, differentiates it from all Alcoota material and that of *Z. trilobus.* The fabellar facet is smaller than that of *Z. trilobus* and more similar in size to diprotodontid fibulae from Alcoota.

#### 3.2.19. Pes

**Figure 24.**
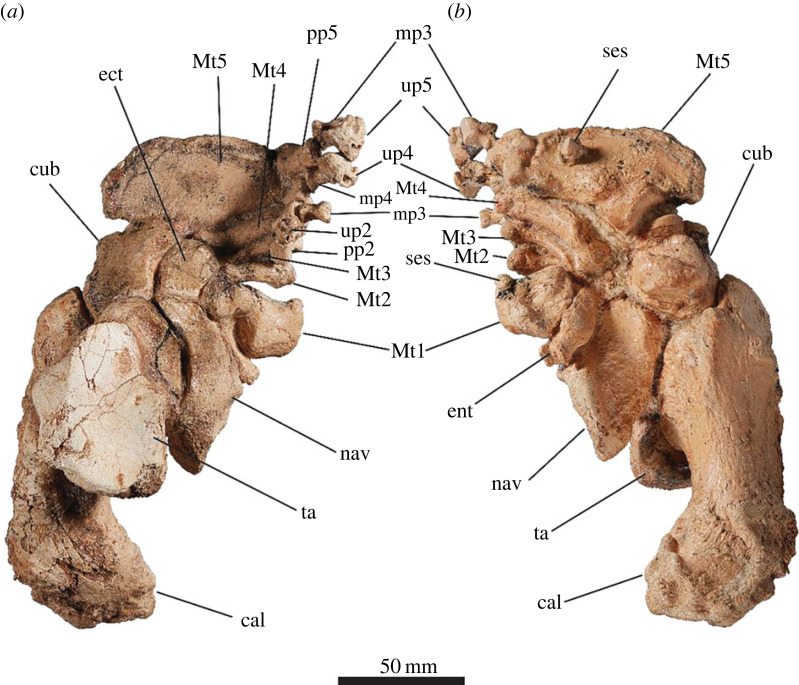
Dorsal and plantar view of left pes of *Ambulator keanei.* Dorsal view (*a*) and plantar view (*b*). Abbreviations: **cal**, calcaneus; **cub**, cuboid; **ect**, ectocuneiform; **ent**, entocuneiform; **mp**, middle phalanx; **Mt**, metatarsal; **nav**, navicular; **pp**, proximal phalanx; **ses**, sesamoid; **ta**, talus; **up**, ungual phalanx.

The pes is angled in such a way that the metatarsals and phalanges curve medially relative to the orientation of the talus, resulting in a crescent-shaped lateral edge (figure 24). The phalanges are curved and directed anterodorsally.

**Figure 25.**
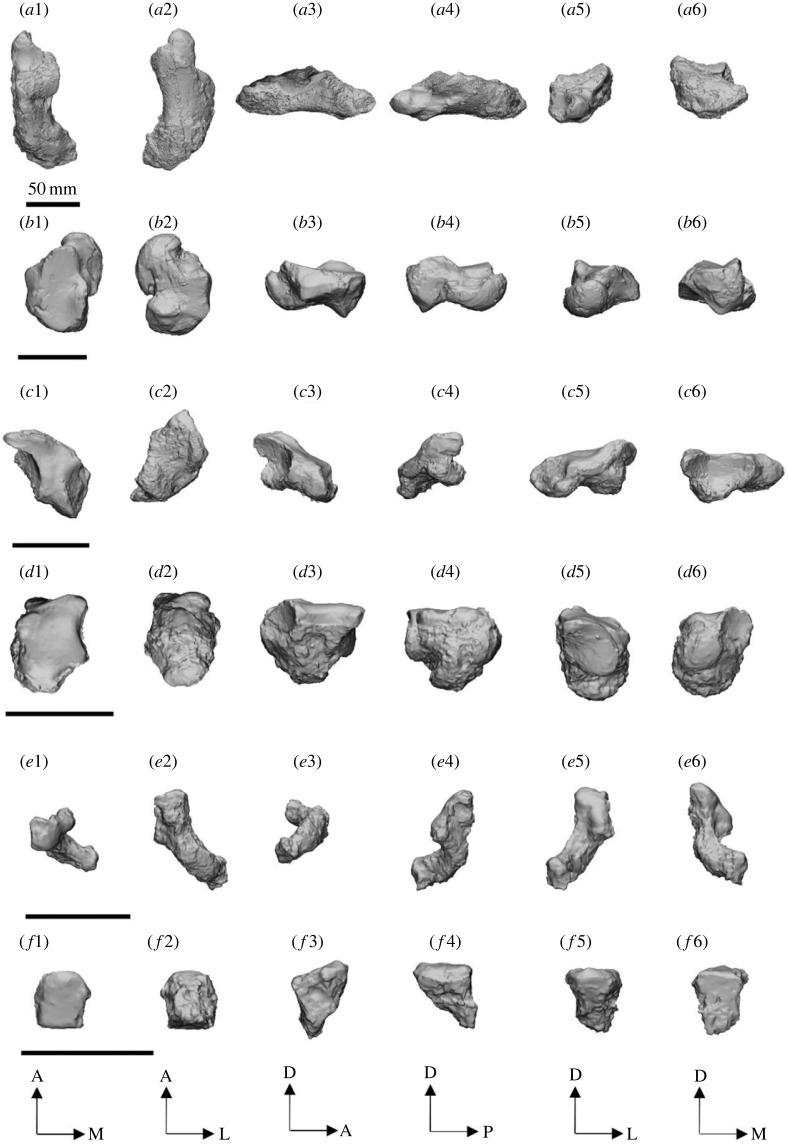
Left tarsals of *Ambulator keanei* (SAMA P54742). Calcaneum (*a*), talus (*b*), navicular (*c*), cuboid (*d*), meso-entocuneiform (*e*) and ectocuneiform (*f*). Dorsal view (1), plantar view (2), medial view (3), lateral view (4), cranial (anterior) view (5) and caudal (posterior) view (6).

The talus (figure 25*b*) is small compared to the rest of the pes. It is located in the centre of the tarsus, is longer than it is wide and dorsoplantarly thick. The tibial facet is oval in dorsal aspect; anteroposteriorly elongated; and is flat, apart from the raised medial crest. The medial crest occupies the posterior half of the medial edge and is concave in lateral view. The fibular facet is 1/5 the size of the tibial facet. The fibular facet is triangular with a plantar point and the flat articular surface is angled dorsolaterally at 125° relative to the tibial facet. The talar neck extends craniomedially from the tibial facet as a depressed surface. The talar head bears the cuboid and navicular facets, which both extend anteromedial of the tibial facet, and is rounded and anteroposteriorly elongate. The cuboid facet occupies 1/4 of the talar head but 3/4 of the cranial face, and has a rounded articular surface; a faint raised border separates it from the navicular facet. The navicular facet is directed medioplantarly and takes up 3/4 of the talar head; its articular surface is convex, which increases the range of possible movement between the talus and the navicular. The calcaneal facet is square with concave edges and a deeply concave articular surface. No clear border is present between the cuboid and calcaneal facets. A large ligamental pit/groove is present between the craniomedial border of the calcaneal facet and the planto-lateral border of the navicular facet.

The talus is small in proportion to the rest of the pes when compared with those of *Z. trilobus*, *D. optatum* and *Euo. grata* (table 3). The tibial facet is oval and takes up a smaller portion of the dorsal surface, unlike the larger rectangular tibial facet in those of *Z. trilobus*, *Euo. grata* and *D. optatum.* The fibular facet is also noticeably smaller than in that of *Euo. grata.* The talus of *Am. keanei* lacks the ligamental fossa on the dorsal part of the talar neck seen in that of *Z. trilobus*. The neck of the talus is more extensive and extends more anteromedially than in those of *Z. trilobus*, *Euo. grata* and *D. optatum*. The navicular facet extends approximately half the length of the talus, as opposed to the 3/4 observed in that of *Euo. grata*. The calcaneal facet is more concave than in that of *Z. trilobus*, resulting in the posteromedial corner of the talus being deeper.

The calcaneus (figure 25*a*) is craniocaudally long, mediolaterally narrow and dorsoplantarly deep and broader at the posterior end. The tuber makes up more than half of the length of the calcaneus and curves medially. The talar facet is L-shaped in dorsal view and widest at the posterior end, covering the entire width of the anterior projection on the medial side, which meets up with the border of the cuboid facet; the articular surface is saddle shaped with a convex caudal portion and concave cranial portion, restricting the craniocaudal movement of the talus. A deep fossa for the calcaneo-talar ligament is caudal to the talar facet. The cuboid facet is a deep, concave depression on the anterior face of the calcaneus, projecting much further on the lateral side. The lateral talar ligamental fossa is shallow and broad. The plantar tubercle is positioned on the lateral face and is anterodorsally elongated, running the length of the tibial facet. The distolateral process on the most anterior point of the calcaneus is pointed from the dorsal aspect and globular on the lateral side.

The calcaneal tuber is most similar to that of *Z. trilobus* but is mediolaterally narrower and dorsoplantarly deeper relative to the length of the calcaneus. The calcaneal tuber is also similar to that of diprotodontid calcaneus NTM P4009 from Alcoota but is longer; however, it is substantially different from the other two morphs observed in Alcoota (e.g. NTM P4008 and NTM P7300). The deep, narrow cross-section of the tuber is reminiscent of those observed in *Pitikantia dailyi*, *Ne. stirtoni* and *Ni. lavarackorum*. The talar facet does not extend onto the distolateral process in contrast with that seen in *Z. trilobus*. Like in that of *Z. trilobus*, the fossa for the calcaneal talar ligament is relatively shallow compared to those observed in *Euo. grata* and *D. optatum*. The cuboid and navicular facets are similar to those of *Z. trilobus* and are plantodorsally shorter than in *Ni. stirtoni*.

The navicular (figure 25*c*) is C-shaped in dorsal aspect, with a large posteromedial process. It articulates with the talus, calcaneus, cuboid, ectocuneiform and meso-entocuneiform. The talar facet takes up approximately 2/3 of the lateral face and is deeply concave, forming a ball and socket joint, allowing some movement between both tarsals. The calcaneal facet is small and located on the cranioplantar edge of the talar facet. The posteromedial process is broadly square and plantarly deflected, resulting in a concave plantar surface; the medial edge is dorsoplantarly narrow and anteroposteriorly elongate with a rounded surface. The meso-entocuneiform facet is thin and extends across most of the anteromedial face, terminating just dorsal of the ectocuneiform facet. The ectocuneiform facet is small, with a flat, rectangular articular surface. The dorsal half of the cuboid facet is a thin surface located on the mediodorsal edge of the navicular between the talar and ectocuneiform facet; the plantar half is rectangular with a flat surface.

The navicular of *Am. keanei* is more similar to those of *Z. trilobus*, *D. optatum* and *Euo. grata* than those of diprotodontids from Alcoota. The talar facet is similar to that of *Z. trilobus* but tapers more posteriorly and is more concave. The cuboid facet was slightly obscured in the CT scan (by articulated elements) but appears to be similar to that of *Z. trilobus,* differing by tapering dorsally to a thinner point, causing the facets for the talus and ectocuneiform to nearly meet. The calcaneal facet was too obscured (by articulated elements) for adequate comparison. The morphology of the medial process is most similar to that of *D. optatum* but has a stronger plantar curve and tapers dorsally; although this does vary in *D. optatum* (e.g. SAMA P58516 and SAMA P5121). The entocuneiform facet is mediolaterally long and dorsoplantarly narrow, similar to those of *D. optatum* and *Z. trilobus*, but extends the same length as the talar facet, as in *D. optatum*. The ectocuneiform facet is also identical to that of *D. optatum*, except in that it nearly comes into contact with the talar facet.

The cuboid (figure 25*d*) articulates with the calcaneus, talus, navicular, ectocuneiform and metatarsal V. It is pentagonal in dorsal aspect with a concave medial edge. The talar facet is oval and deeply concave. The navicular facet is positioned craniomedially to the talar facet; the lower half is flat and is approximately half the size of the talar facet while the upper half is a thin surface between the talar and ectocuneiform facets. The calcaneal facet is positioned plantolateral to the talar facet and is around the same size; it is circular and convex. The plantar tuberosity is large, rugose and hooks anteriorly. A shallow groove is present on the plantolateral face of the cuboid between the plantar tubercle and the calcaneal facet. A deep groove is present between the plantar tuberosity and the metatarsal 5 facet. It is possible that either of the previous grooves may provide passage for the *m. peroneus longus* tendon. The lateral tuberosity extends along the lateral edge of the cuboid. A smooth surface on the anterior face of the lateral plantar tuberosity is present. The ectocuneiform facet is triangular and deeply craniocaudally concave. The metatarsal 5 facet is oval and saddle-shaped.

The cuboid of *Am. keanei* is more similar in shape to that of *Z. trilobus* than any other compared diprotodontid. The talar facet is more concave, and smaller than in that of *Z. trilobus*. The medial plantar tubercle is larger than in that of *Z. trilobus*, similar to that in the Alcoota diprotodontid (NTM P7286) but is more anteriorly hooked, as in that of *D. optatum*. The lateral tubercle extends anteroposterior, as in that of *Euo. grata*. The ectocuneiform facet is more concave than in that of *Z. trilobus*. The metatarsal 5 facet is smaller and more concave than in that of *Z. trilobus*.

The ectocuneiform (figure 25*f*) is the smallest of the tarsals. It is pyramidal, narrowing to a plantar point. It articulates with the cuboid, navicular, meso-entocuneiform and metatarsals 2, 3 and 4. The navicular facet takes up the entire posterior face, the flat articular surface restricting rotational movement. The mesocuneiform facet takes up the entire medial face; its surface is slightly concave, allowing for some rotational movement. The cuboid facet takes up the majority of the lateral face; it is split into a dorsal and plantar facet that is separated by the cuboid-ectocuneiform ligamental fossa in the centre. The anterior face is slightly pointed causing metatarsal 2, metatarsal 3 and metatarsal 4 to be angled medially from the sagittal plane. The facet for metatarsal 4 is the largest of the three metatarsal facets on the anterior face and is restricted to the dorsal section of the ectocuneiform. The facet for metatarsal 3 is small and triangular and is the second smallest facet on the anterior face. The metatarsal 2 facet is a very small triangular facet located on the anteromedial corner.

Due to the poor resolution of surfaces in the CT scan, detailed comparisons of the articular facets are not possible; the specimen is bound together by a hard calcareous matrix, which prohibits disarticulating the specimen without damage and loss of preserved relationships. Given that the morphology of the articular facets appears to vary between specimens of *D. optatum* (UCMP 5735 (right), SAMA P5121, UCMP 57350 (left)), precise details of their shape may not be important. Their overall morphology appears to be more similar to that seen in *D. optatum* than that in the ectocuneiforms NTM P2999, NTM P2998 and NTM P2997 from Alcoota.

The entocuneiform (figure 25*e*) is fused with the mesocuneiform. The combined tarsal is thin and mediolaterally elongated; it is dorsoplantarly concave; and is most robust at the medial end. It articulates with the navicular, hallux, metatarsal 2 and the entocuneiform. The navicular facet is elongated and extends across the entire posterior face. The entocuneiform facet is small and flat. The CT scan does not reveal details of the metatarsal 2 facet but it appears to be small and located on the dorsolateral corner of the anterior face. The hallucal facet is deeply mediolaterally concave and occupies most of the anterior face. Two tubercles, one dorsal and the other plantar, protrude from the medial extent of the body.

The entocuneiform of *Am. keanei* differs from those of all diprotodontid entocuneiforms from Alcoota by being fused to the mesocuneiform. It differs from that of *Euo. grata* by not also being fused with the ectocuneiform. Is more similar to that of *Z. trilobus* than that of *D. optatum.* The body is craniocaudally narrower relative the mediolateral length than in those of *Z. trilobus,* and the metatarsal 1 facet is larger and more concave. The plantar and dorsal tubercles on the medial extremity of the body are found in NTM P4005, NTM P4006 and NTM P4007 from Alcoota, but are more prominent in *Am. Keanei*.

**Table 3.** Comparative pedal measurements for *Ambulator keanei*, *Euowenia grata* and *Diprotodon optatum*. L = length, W = width.

species	*Ambulator keanei*				*Zygomaturus trilobus*		*Euowenia grata*		*Diprotodon optatum*	
specimen	SAMA P54742		SAMA P52377		TMAG Z3554		SAMA P49922		SAM P5121	
	L	W	L	W	L	W	L	W	L	W
talus	75.9	54.9			97.2	81.9	93.0	73.2	139.3	94.0
calcaneus	126.5	44.7	143.1	62.6	—	—	158.7	61.7	196.7	87.7
navicular	43.7	64.8	—	—	—	87.2	61.2	89.8	62.6	110.7
cuboid	52.4	36.9	—	—	50.0	39.5	42.2	48.5	73.0	72.6
ectocuneiform	21.5	22.0	—	—	—	—	—	—	40.6	31.3
entocuneiform	30.3	55.9	—	—	—	—	—	—	28.0	87.7
metatarsal 1	29.4	28.5	—	—	—	—	32.0	17.7	52.5	32.3
metatarsal 2	31.9	7.4	—	—	52.2	15.2	35.3	13.9	29.8	10.2
metatarsal 3	37.1	6.8	—	—	—	—	44.3	7.8	51.3	10.2
metatarsal 4	51.3	15.1	—	—	66.2	—	56.5	12.6	66.3	22.0
metatarsal 5	75.1	45.7	86.9	42.3	—	—	83.1	49.7	107.8	75.2
proximal phalanx 2	19.3	6.9	—	—	—	—	19.8	9.8	21.0	9.7
proximal phalanx 3	18.5	7.1	—	—	—	—	20.9	9.9	22.1	8.5
proximal phalanx 4	17.4	9.5	—	—	—	—	21.7	13.0	26.2	18.2
proximal phalanx 5	21.0	24.5	—	—	—	—	24.2	16.0	33.2	25.6
middle phalanx 2	—	7.3	—	—	—	—	12.8	8.7	14.7	10.7
middle phalanx 3	11.1	5.7	—	—	—	—	12.4	9.9	19.5	9.8
middle phalanx 4	10.0	6.5	—	—	—	—	13.0	10.1	16.9	14.3
middle phalanx 5	11.9	9.4	—	—	—	—	14.0	12.4	21.9	22.0
ungual phalanx 2	—	—	—	—	—	—	—	7.7	24.8	12.8
ungual phalanx 3	—	—	—	—	—	—	27.4	7.8	30.5	20.3
ungual phalanx 4	—	7.3	—	—	55.9	23.7	28.6	8.2	31.9	12.4
ungual phalanx 5	—	10.9	—	—	—	—	27.9	10.4	37.2	14.8

The hallux (metatarsal 1) is square in dorsal view, 25.5 mm/30.0 mm, articulating with the entocuneiform and rotated in the transverse plane. The plantar face is concave with a small ligamental fossa in the centre. The entocuneiform facet is anterodorsally concave. A small ligamental fossa is present on the plantar face. A large, rounded sesamoid on the proximal anterior corner of the plantar face may indicate the presence of a phalanx that has been disarticulated and separated; although this sesamoid may have shifted from another location on the pes throughout the taphonomic process, since this is not observed in any other diprotodontid.

Metatarsal 2 is the smallest of the five metatarsals and articulates with the ectocuneiform, mesocuneiform and metatarsal 3. It is cylindrical, being widest on the distal end and tapers proximally. The proximal articular end is triangular in dorsal view and composed of three facets. The mesocuneiform facet is on the posterior side and is the largest of the three. The entocuneiform facet is the smallest and is situated on the proximal-most tip. The metatarsal 3 facet is on the cranioproximal edge. The distal articular surface is globular.

Metatarsal 3 is the second smallest metatarsal; it is cylindrical in overall shape and widest distally, and tapers proximally. It articulates with the entocuneiform, metatarsal 4 and metatarsal 2. The distal articular surface is globular.

Metatarsal 4 is cylindrical and widest distally, and tapers proximally. It articulates with the entocuneiform, metatarsal 5 and metatarsal 3. The distal articular surface is globular with a slight keel on the plantar surface.

Metatarsal 5 articulates proximally with the cuboid and metatarsal 4. It is relatively flat and triangular in dorsal view with a convex lateral edge, similar to metacarpal 5, and curves medially to take up approximately half the anterior surface area of the pes. The cuboid facet is circular and mediolaterally concave. A large process for attachment of the *mm. peroneus* extends laterally from the cuboid facet. The distal articular surface is globular with a flat plantar surface.

A lateral pedal digital sesamoid is lateral of the distal articular facet. It is large, rounded and articulates with metatarsal 5 and proximal phalanx 5. The other sesamoid that would be on the medial side of the distal articular facet of metatarsal 5 has been displaced to the centre of the plantar surface of the metatarsal 5 (figure 24).

Metatarsal 5 is most similar to that of *Z. trilobus,* differing from those of *D. optatum* and *Euo. grata* by the lack of distinctly separate insertion points for the *mm. peroneus* on the lateral edge of the metatarsal 5. The metatarsal 2 is straighter and less arched than in those of *D. optatum* and *Euo. grata*. The metatarsal 1 is wider than in those of *D. optatum* and *Euo. grata.* The distal articular processes on metatarsal 2 to metatarsal 5 differ from those of *Ng. tedfordi*, *Ni. lavarackorum*, *Ne. stirtoni* and all Alcoota diprotodontids, except NTMP 4991 by the absence of a keel for sesamoids.

Four digits project craniomedially from the craniodorsal distal articular facets of metatarsals 2–5 in the pes. All proximal phalanges are widest at the proximal end before tapering to a waist at 2/3 their length distally and then widen at the distal articular facet. The distal articular facets are saddle shaped with the articular surface being dorsoplantarly concave and slightly mediolaterally concave. Proximal phalanx 5 is the largest, with proximal phalanx 3 being the smallest and 2 and 4 are approximately equal in size; this same pattern applies to the middle phalanges. The plantar faces of proximal phalanges 5 and 4 have grooves for flexor tendons and this groove is largest in phalanx 5. The distal articular surfaces are only slightly rounded, allowing for little movement between the proximal and middle phalanges and restricting them to a permanently partially flexed position.

The middle phalanges are shorter than the proximal and ungual phalanges. All middle phalanges are cylindrical (except the fifth) and constricted in width at mid-length. The proximal articular surfaces are oval and mediolaterally wide, shallowly concave, and form a weak ball and socket joint with the proximal phalanges. The distal articular facets are barrel shaped with the surface being dorsoplantarly slightly concave.

Ungual phalanges 5 and 4 are present in SAMA P54742, with the distal ends broken off. Both have large, arch-shaped articular facets. The surface is saddle shaped and slightly raised at the medial keel. A flexor tubercle is present on the proximoplantar end of ungual phalanges and is largest in ungual phalanx 5. The flexor tubercles are triangular in plantar view, tapering anteriorly; a deep fossa is present on the medial and lateral sides. Both unguals are broken at the distal ends making the shape of the unguis difficult to determine.

Phalanges of *Am. keanei* differ from those of *Euo. grata* by being more robust; the distal articular surfaces of the proximal phalanges are less plantarly deflected; the articular facets for sesamoids are larger in proximal phalanges 5 and 4; and a distinct median keel is present on the unguals. A large sulcus is present on the proximal phalanx 5. Phalanges of *Am. keanei* differ from those of *D. optatum* by a large groove for flexor tendon on proximal phalanx 5.

#### 3.2.20. Footpad

**Figure 26.**
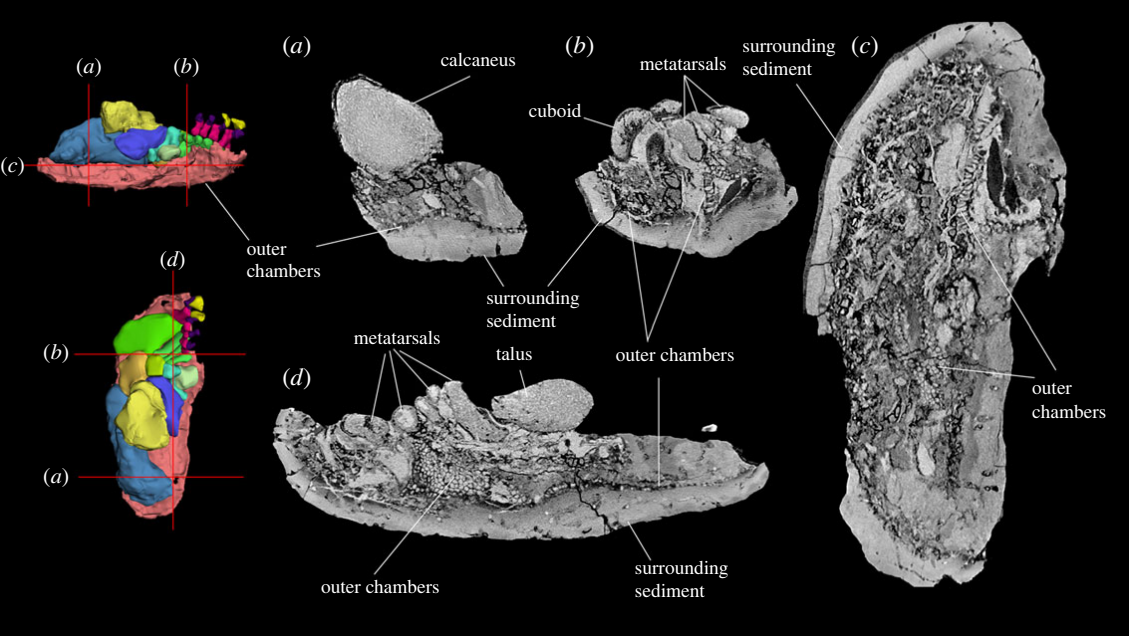
Cross-sectional images from the CT scan of the left pes of *Ambulator keanei* (SAMA P54742) (not to scale).

The description of footpad morphology is based on observations of the images derived from the CT scan (figure 26). The CT scan of the pes revealed structures resembling small chambers plantar to the podials. These structures appear to be consistent with the adipose tissue observed in the pes of elephants and humans [58,59]. Their form is consistent with the structure of unpublished *D. optatum* footpad impressions from Lake Callabonna (A.B.C. and T.H.W., personal observation) and therefore are interpreted as fossilized/replaced soft tissue structures that may have been replaced during rapid anaerobic decay of organic matter [60], more specifically the surrounding collagen fibres and septa of the adipose tissue [58,59]. Therefore, we describe this potential soft tissue here.

**Figure 27.**
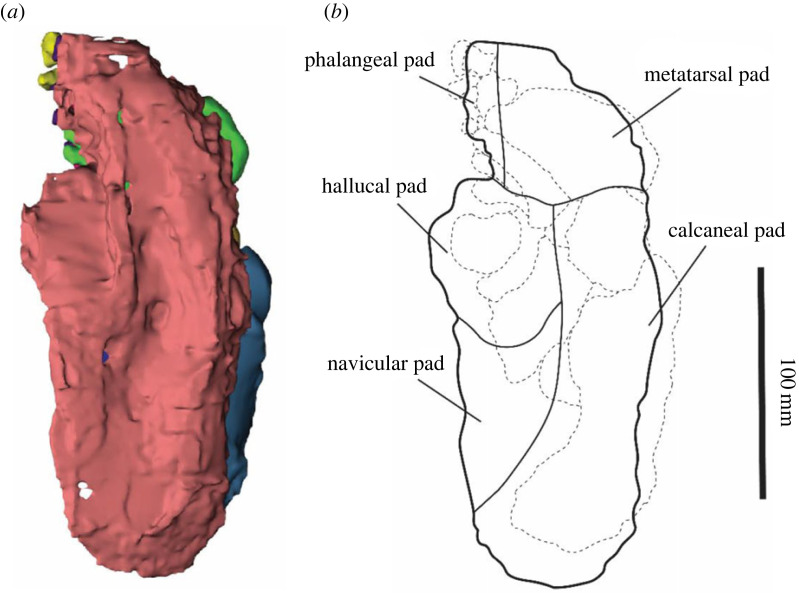
Left pes footpad of *Ambulator keanei* (SAMA P54742). Segmented footpad from CT scan in red (*a*). Interpretation of footpad structure with underlying bone is illustrated with dotted lines (*b*).

The outer perimeter of the footpad impression is oval in overall shape (in plantar aspect) with five distinguishable sections which we refer to as pads (figures 26 and 27); while the exact borders between these pads were difficult to deduce, we provide one interpretation for them in figure 27, which is based on abrupt changes in the outer layer of chamber structure and position. The outer structure of the footpad is formed of the pseudomorphs of chambers that may have been the adipose tissue. They enclose a series of cavity structures that may have been larger chambers that can be observed between the bone and the outer dermal layer. The deepest is below the calcaneus and metatarsal 5. The plantar face of the calcaneal pad was populated with globular dermal papillae that were dorsoplantarly compressed at the junction between the phalangeal and metatarsal pads. A large groove, a crease between pads, is present between the medial border of the tarsal pad and the navicular pad. The outer chambers plantomedial of the calcaneus within the groove are oval, dorsoplantarly elongated in the anterior and lateral cross-section and circular in the dorsal cross-section.

The navicular pad is medial to the calcaneal pad and terminates below the proximal half of the navicular, but most of the pad sits between the posterior border of the navicular and the medial curve of the calcaneal tuber. The plantar surface is populated with dorsoplantarly elongate outer chambers. A majority of the plantar surface is plantarly convex, as is the calcaneal pad.

The hallucal pad is rectangular and rose abruptly dorsally of the navicular pad. The outer chambers were plantar-dorsally elongated.

The metatarsal pad is the second largest pad, making up the entire anterolateral border of the pes. It extends unbroken from under the posterior border of the calcaneus to the distal end of metatarsal 5 and is sickle shaped. The outer chambers are oval and mediolaterally elongated in the anterior cross-section. The plantar surface of the footpad rises dorsally at near 90° from the plantar surface of the metatarsal pad below the border of the phalanges and metatarsal.

The dermal papillae on the phalangeal pad are closest to the bones of the foot and nearly contacting the phalanges. The outer chambers are oval in dorsal and anterior cross-sections (figure 22*b*,*c*).

### 3.3. Body size estimates

The mean body size estimate of *Am. keanei* is 262 kg, with an upper bound of 334.1 kg and a lower bound of 190.0 kg.

## 4. Discussion

**Figure 28.**
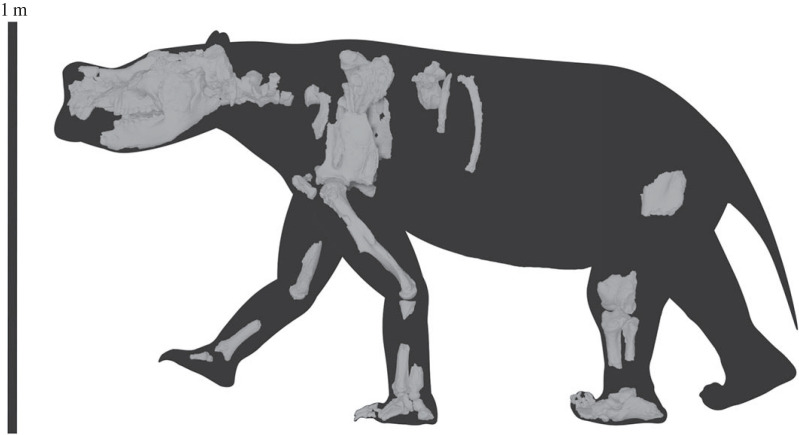
Reassembled partial skeleton *Ambulator keanei* (SAMA P54742) with silhouette.

Prior to this study, *Ambulator keanei* was described solely from tooth-bearing portions of the skull and isolated teeth. The partial skeleton described here (figure 28) represents the most complete known specimen of *Am. keanei*, and the most complete known for any Plio-Pleistocene diprotodontoid other than *Diprotodon optatum* and *Zygomaturus trilobus*. Consequently, it affords the opportunity to assess its systematics, body size and dietary and locomotory adaptations, which will help enable more robust phylogenetic and palaeoecological analyses once similar analyses of other key diprotodontid taxa are completed.

### 4.1. Systematic implications

#### 4.1.1. Comparison with *Zygomaturus*

Historically, an overwhelming emphasis has been placed on dental morphology to inform diprotodontid taxonomy, with little attention paid to the rest of the skeleton [6,11,61]. This was because teeth are among the hardest components in the body, thus, the most likely to be fossilized, described and used as a basis for defining species. Particular attention has been paid to P3 morphology [6–14]. However, recent studies have reported on the marked intraspecific variability in the premolar of well-represented diprotodontid species and have raised concerns of an overreliance on these teeth [62,63]. We also found high intraspecific variability in premolar morphology, such that few morphological features were consistently different between *Am. keanei* and *Z. trilobus*. No consistent differences between specimens of *Am. keanei* and the holotype of *Z. gilli* (NMV P209962) were found (figure 3).

The variability of the premolar in *Z. trilobus* was originally considered by Stirton [64] to be too great to indicate that the incomplete P3, NMV P209962, should be used as a holotype for a new species of *Zygomaturus*. He later reconsidered and made it the holotype of *Z. gilli* [10]. Here we support Stirton's original conclusion and recommend that *Z. gilli* be considered a *nomen dubium*; the morphology of the holotype P3 cannot be distinguished from those of *Am. keanei* or *Z. trilobus*. We recognize that some material (NMV P16279 and NVM P15911) from the type locality of *Z. gilli* (Beaumaris, Victoria) differs sufficiently from that of *Am. keanei* to warrant specific distinction (e.g. Rich *et al*. [65]); however, a taxonomic review of the Beaumaris material is beyond the scope of this study.

There are also few characters of the molar dentition that can consistently be used to distinguish some diprotodontid taxa, including *Am. keanei*, *Z. trilobus* and *Hulitherium tomasettii.* Yet these taxa differ markedly in cranial and limb morphology. Some characters that were previously hypothesized to be diagnostic for *Am. keanei*, such as the mesostyle [10], vary intraspecifically. It was thought that the absence of the mesostyle, combined with its smaller size and other minor differences in the molars, could suggest that the Lawson–Daily Quarry specimen (UCMP 70126) belonged to a separate species [10]; we advocate that these differences are the product of intraspecific variation with *Am. keanei*. Our observations suggest that caution should be implemented when identifying diprotodontid taxa to species level from the dentition alone.

Undescribed postcranial elements are known for a range of diprotodontids (*Maokopia ronaldi*, *Euryzygoma dunense*, *Meniscolophus mawsoni*, *Kolopsis torus*, *Pyramios alcootense* and *Plaisiodon centralis*). The postcranial material of the Alcoota diprotodontids was referred to as a collective here, but future studies should endeavour to find element associations within the sample that can be tied to the craniodental remains from which Alcoota species have hitherto been named. A method could be to associate the elements through phylogenetic inferences for other taxa, i.e. associating more material with features only found in diprotodontines or zygomaturines. However, the higher level systematics of the Diprotodontidae is in urgent need of review, particularly with the inclusion of postcranial characters. Another method may be to associate elements by size, abundance and functional groups. Postcranial material from the Lawson–Daily Quarry may belong to *Me. mawsoni*, since it does not appear to belong to *Am. keanei* or *Euo. grata,* which are both found in the same deposit (personal observation). Through our comparisons, we found that a humerus (NMV P232193) ascribed to a species of *Zygomaturus*, from Batesford Limestone Quarry, was slightly different in the termination of the deltopectoral crest from those of *Am. keanei* and *Z. trilobus*; yet, the dentition from Batesford Limestone Quarry could not be differentiated from those of *Am. keanei* and *Z. trilobus*. The specimens were found from an *in situ* bedded stratum of the Moorabool Viaduct Sand; 2.2 m above the underlying Fyansford Clay and Moorabool Viaduct Sand (E. Fitzgerald 2023, personal communication). The excavation of more material may lead to a new species being described from the deposit.

#### 4.1.2. Higher level taxonomy

Higher level taxonomy of the Diprotodontidae is also in urgent need of re-assessment. Due to the incompleteness of the fossil record, and the overriding focus on dental remains by mammalian palaeontologists, many diprotodontid genera have been diagnosed solely on dental features [10,22,66]. Still, some have also been diagnosed on osteological characteristics, such as the forms of the masseteric process of *Euryzygoma* [67] and the femur of *Hulitherium* [15]. The genus *Meniscolophus* was described as a new genus because much of the comparative material now available did not exist at the time, viz. ‘we do not have enough information on the types and other specimens in museum collections to determine the magnitude of these differences' [22, p. 258]. *Alkwertatherium webbi* has very few differences from the diprotodontine *Pyramios alcootense*, but it differs by having a parastyle on P3, which has usually been seen as characteristic of the Zygomaturinae, not the Diprotodontinae [9].

The stability of other genera is also in need of reassessment. For example, *Kolopsis* has been suspected to be polyphyletic [68], while *Me. mawsoni* and *Eur. dunense* have been considered to possibly be congeneric [14]. We suspect a comprehensive phylogenetic analysis that includes cranial and postcranial material may resolve the phylogenetic position and validity of many of these genera. The skeletal morphologies of *Am. keanei* and *Z. trilobus* are substantially different, with few overlapping features in the skull and limbs. Furthermore, many of the overlapping features may be a result of increased adaptation for graviportality (adaption to slow quadrupedal locomotion and a high body weight), because they are shared with *D. optatum* and *Euo. grata*. Leaving *Am. keanei* within *Zygomaturus* would also effectively mean that the genus would include other diprotodontids such as *Hulitherium tomasettii*.

### 4.2. Craniodental morphology and diet of *Ambulator keanei*

The dental morphology of *Am. keanei* is very similar to that of *Z. trilobus*, which led to its previous placement in the same genus. However, the cranium is markedly different. It is more elongate and most similar overall to that of *Kolopsis torus*. The incisors of *Am. keanei* consist of a large I1 with a smaller I2 and I3, unlike the broad, chisel-shaped I1 of *D. optatum* or the wide I3 and I2 of *Neohelos stirtoni*. Herbivores that consume large amounts of tougher, low-nutrient food (e.g. grazers) usually possess broader incisors and a larger incisor arcade, while those that eat lower quantities of softer, more nutritious food (browsers) usually have smaller, narrower, more selective incisors [69,70]. Consequently, *Am. keanei* may have consumed a higher proportion of browse than *D. optatum*, which is hypothesized to have been a mixed feeder [71–73]. The lower incisors of *Am. keanei* are splayed and pointed similar to those of *Z. trilobus*, not scooped as in *Py. alcootense* or bladed as in *Kolopsis torus*, and may have been less suitable for cutting and procuring food. This tusk-like lower incisor morphology may also have had a secondary function, e.g. in defence or intraspecific competition, similar to *Rhinoceros unicornis* (Indian rhinoceros) [74,75]. It is possible they may have also procured food items though the aid of prehensile lips, such as those of some extant large herbivores [76].

The molars of *Am. keanei* are low crowned, bilophodont, and characterized by wide interlophid valleys and low crests, similar to those of *H. tomasettii* and *Z. trilobus,* but unlike the high-crowned molars of *D. optatum*. Previous analyses of molar microwear and *δ*^13^C isotopes of both *H. tomasettii* and *Z. trilobus* have shown values expected in those of browsers [73,77]. However, low *δ*^13^C values typical of browsers have also been observed in dental material from *Eur. dunense*, which has a very different molar morphology [78]. Higher crowned molars usually characterize extant taxa that consume more abrasive foods, such as grasses [69,79–81]. By comparison with its contemporaries *Eur. dunense*, *Me. mawsoni* and *Euo. grata*, the lower crown height of *Am. keanei* may indicate that it was adapted to a less abrasive diet. The extremely worn teeth of SAMA P54742 may, for this individual, reflect old age or a high-wear diet to which *Am. keanei* was not expressly adapted. In addition, the smaller crests and wider interloph(id) valleys compared with those of *Eur. dunense*, *Me. mawsoni* and *Euo. grata* may have provided a greater surface area for crushing and grinding food than cutting, which is also consistent with less abrasive food [69,82].

The smaller zygomatic arch and masseteric process in *Am. keanei* would have provided less attachment area for the masseter than in *Z. trilobus*, *Euo. grata* and *Eur. dunense.* The size and proportions of jaw adductor muscles have been shown to differ between different dietary groups of extant marsupial herbivores [83,84]. Expanded masseter muscles are typically correlated with a large transverse chewing component, which is essential for shearing and grinding tough, fibrous material that requires a lot of processing [83,85]. Masseter muscles positioned above the tooth row can also assist in crushing hard food items [86,87]. The attachment areas for the masseter muscles in *Am. keanei* are proportionally smaller compared to the cranium and positioned more posterior to the tooth row than to those of *Z. trilobus*. This suggests that *Am. keanei* primarily fed on softer material that required less processing than the diets of *Z. trilobus* and *D. optatum*.

### 4.3. Locomotion

#### 4.3.1. Forelimb

The forelimb of *Am. keanei* is morphologically more similar to that of *D. optatum* than to those of *Z. trilobus*, *Ne. stirtoni*, *Ng. tedfordi*, *H. tomasettii*, *Kolopsis torus*, *Pl. centralis* and *Py. alcootense*. The humeral head of *Am. keanei* is more ovoid and less caudally projected than that of *Ne. stirtoni*. This is thought to stabilize the shoulder and reduce movement to the anteroposterior plane during locomotion [88,89]. A less projected humeral head may also be associated with a larger proportion of the body mass supported by the forelimb and differs between larger and smaller species of rhinoceros [90]. The large attachment sites for the *m. latissimus dorsi* and *m. deltoideus* in *Am. keanei* indicate restricted adduction and abduction of the arm. The large *m. deltoideus* attachment site also suggests the deltoids acted to extend and stabilize the shoulder during locomotion.

The forelimb morphologies of Pleistocene diprotodontids and *Am. keanei* superficially resemble that of the *Loxodonta africana* (African elephant), as Murray [91] observed for *D. optatum*. The prominent infraglenoid process observed in the scapulae of *Am. keanei*, *Z. trilobus* and *D. optatum* would provide a large attachment area and leverage for the *m. triceps brachii caput longum*, which originates in this area in other marsupials [39,40,42] (unpublished dissections of *Lasiorhinus latifrons* (southern hairy-nosed wombat) and *Phascolarctos cinereus* (koala) by J.D.v.Z. and A.B.C.). The structure of the infraglenoid tubercle resembles the hooked angulus caudalis in *Lo. africana*, which provides an attachment area for a range of muscles, including the *m. triceps brachii caput longum* [92].

The robust, medially deflected cranial angle of the scapula (also reminiscent of *Lo. africana*) provides attachment for the *m. rhomboids* and likely was antagonistic to the *m. triceps brachii caput longum*. The large, caudally hooked coracoid process provides a larger lever for the *m. biceps brachii*, which may have been important for weight bearing by preventing flexion of the shoulder joint and countering the *m. triceps* while standing, similar to that in extant horses [93]. These features are present in *Am. keanei*, *Z. trilobus* and *D. optatum*, but absent from late Miocene taxa (e.g. *Kolopsis torus*, *Pl. centralis*, *Py. alcootense*). They suggest that *Am. keanei* was capable of strong extension of the elbow and shoulder to support its weight during locomotion. Similarities in forelimb morphology to *Lo. africana* have previously been hypothesized by Murray [91] to suggest that *D. optatum* (and by extension *Am. keanei*) may have walked by swinging its forelimbs in a pendulous, low-energy form of locomotion. This is supported by trackway evidence of Pliocene and Pleistocene diprotodontids, where the footprint for the manus and pes overlap [94,95].

The morphology of the distal humerus of *Am. keanei* is similar to that of *Z. trilobus* indicating a similar columnar posture and range of motion [49]. The supracondylar bridge may function to protect the ulnar nerve and brachial artery during elbow flexion and, consequently, its presence may indicate a more flexed natural position of the elbow [96,97]. However, its presence in *Am. keanei* may be of phylogenetic importance due to its presence in zygomaturines *(Z. trilobus*, *Ni. lavarackorum* and *Ne. stirtoni*) and absence from Pliocene and Pleistocene diprotodontines (*D. optatum* and various humeri that could be *Eur. dunense* or *Euo. grata*). The proximal ulna is not known for *Am. keanei*.

The manus of *Am. keanei* appears to be primarily adapted to weight bearing as opposed to other functions such as grasping. The large styloid process on the ulna and hamatum process on the radius indicates more weight being distributed across the manus than in earlier diprotodontids (*Ni. lavarackorum*, *Ne. stirtoni*, *Ng. tedfordi*, *Kolopsis torus*, *Pl. centralis* and *Py. alcootense*). The large ball and socket joints (radius-scaphoid and ulna-pisiform-triquetrum) also provide additional bracing against lateral stresses when compared to pre-Pliocene diprotodontids (*Ne. stirtoni*, *Ng. tedfordi*, *Ni. lavarackorum*, *Kolopsis torus*, *Pl. centralis* and *Py. alcootense*). The large size of the carpals relative to the metacarpals suggests that a substantial portion of the weight-bearing role has been shifted from the phalanges to the carpus, including a significant portion of weight on the pisiform, as seen in *D. optatum* (figure 28) and, to a lesser extent, *Z. trilobus*. Pleistocene diprotodontids have a unique form of weight bearing where the pisiform has rotated caudally and acts in a support role (heel) similar to the calcaneus in the pes [17,97]. This adaptation is absent in earlier diprotodontids (*Ne. stirtoni*, *Ng. tedfordi*, *Ni. lavarackorum*, *Kolopsis torus*, *Pl. centralis* and *Py. alcootense*), making *Am. keanei* the earliest diprotodontid species observed to possess it (figure 18). The orientation of the pisiform in *Am. keanei* is not yet fully horizontal, as is seen in *D. optatum.* The large pisiform also indicates a large attachment area for the *m. flexor carpi ulnaris* tendon that has been modified to function similarly to the *m. gastrocnemius* tendon in the hindlimb. The pisiform may also function to stabilize the manus, similar to the praepollex in elephants [98]. This may suggest *Am. keanei* was better adapted to a more energy efficient plantigrade locomotion than earlier diprotodontid taxa, similar to plantigrade adaptations observed in polar bears and humans [99,100].

The hamate process of the hamatum provides attachment for the flexor retinaculum, guiding the flexor tendons of the digits. This process is smaller in *Am. keanei* than in the Alcoota diprotodontids (*Kolopsis torus*, *Pl. centralis* and *Py. alcootense*), but larger than in *D. optatum* or *Z. trilobus* (figure 28), suggesting a smaller flexor tendon volume than in the manii of Alcoota diprotodontids*.* The lack of a strong keel on the palmodistal metacarpals suggests relatively small sesamoids and little lateral stress on these joints. This points to a relatively weak ability to flex the digits compared to earlier diprotodontids and *Am. keanei* is similar to *D. optatum* in this respect (figure 20). This, combined with the flat proximal articular surface between the proximal and middle phalanges, means there was little movement possible for flexion and extension of the digits. By articulating the manus, we hypothesize that the phalanges may have been slightly raised off the ground and not weight bearing during locomotion (figure 18), perhaps also facilitated by a large manual pad beneath the carpals and metacarpals. This was also suggested for *D. optatum* by Weisbecker & Archer [101] and is further supported by the lack of manual digit imprints in trackways [95,97]. The ungual phalanges are not as flat as they are in *D. optatum* or in the more extreme *Z. trilobus*. They likely served little purpose during locomotion.

The overall morphology of the forelimbs of *Am. keanei* appears to reflect adaptation to graviportal, quadrupedal locomotion, with more overall similarity to the forelimbs of *D. optatum* and *Z. trilobus* than to any pre-Pleistocene diprotodontid.

#### 4.3.2. Hindlimb

The hindlimb of *Am. keanei* is more similar to that of *D. optatum* than any other diprotodontid. The proportionally smaller femoral facet on the fibula compared to other diprotodontids suggests it plays a lesser role in weight bearing. The medial trochlear crest is proportionally larger than in any other diprotodontid known, suggesting a greater need to stop the patelloid slipping medially, indicating a more sprawled stance. Ungulates with increased asymmetry in the distal femoral condyles tend to occupy more open habitats [102].

The condition of having an inwardly rotated pes (relative to the direction of the talar facet) with an enlarged metatarsal 5 and calcaneus is superficially reminiscent of that seen in ground sloths, in which it has been termed ‘pedolateral rotation’ and hypothesized to allow for their upright bipedal position [103]. The outer layer of chambers ventral to the bones appears to be consistent with structures of adipose tissue in those of elephants and humans [58,59,104,105]. Within the footpads, between the outer adipose chambers and the bone may have been enlarged fat chambers that presumably functioned as shock absorbers for weight bearing as they do in elephants and humans [58,59,104]. The distance between the bone and the outer adipose tissue is greatest below the calcaneus and metatarsal 5 footpads in the *Am. keanei* material, close to where the pressure is greatest in humans ([58] and references therein), suggesting more weight was being placed there. The talus is proportionately smaller relative to the rest of the pes than in other diprotodontids such as *D. optatum*, *Euo. grata* and *Z. trilobus,* which may reflect less weight being placed on the hindlimbs relative to the forelimbs. Unlike in the manus, some weight-bearing adaptations are present in the pes of the Miocene diprotodontids. The calcaneus is elongated and the tuber is curved to brace against lateral stresses that may result from its large body mass and wide gait. The large plantar tuberosities on the calcaneus and cuboid provide extra muscle attachments for the *m. gastrocnemius* and tendinous masses for flexion of the foot during locomotion.

**Figure 29.**
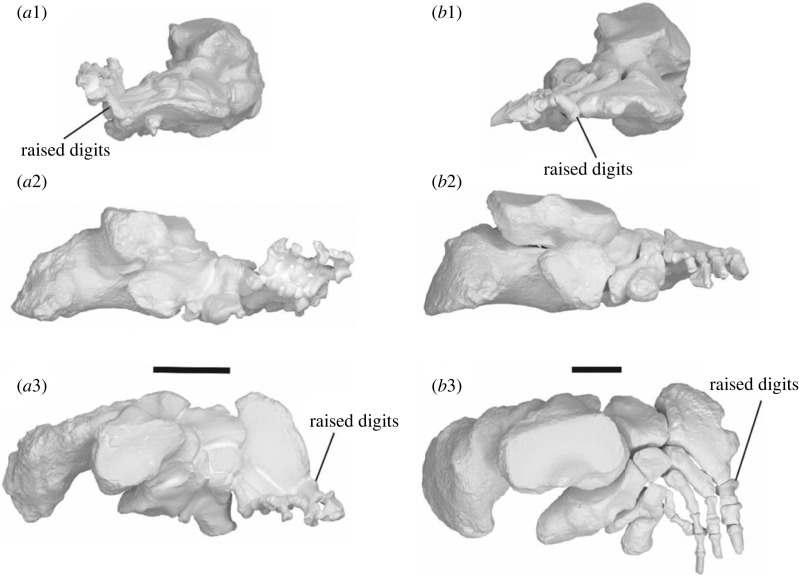
Left pes of *Ambulator keanei* (SAMA P54742) (*a*), right pes (mirrored) of *Diprotodon optatum* (*b*). Anterior view (1), medial view (2) and dorsal view (3).

The orientation of the articulation between the middle and proximal phalanges suggests elevation and foreshortening of the digits, more so than in the manus, perhaps to keep the gracile phalanges from breaking during locomotion, or they may just be vestigial. As in *Euo. grata*, the phalanges of *Am. keanei* were perhaps useful for grooming and served no real function for locomotion [16]*.* On closer inspection, this feature is shared between *D. optatum*, *Euo. grata* and perhaps even *Z. trilobus*. Consequently, as an alternative to the flat position of the pedal phalanges that appear in prior reconstructions of the pes [17], we propose that they were instead raised, perhaps to avoid injury (figure 29).

### 4.4. Palaeoenvironment and evolutionary significance

Many aspects of the postcranial morphology of *Am. keanei* have greater similarity to those of *Z. trilobus* and *D. optatum* than they do to earlier diprotodontids such as *Kolopsis torus*, *Pl. centralis* and *Ne. stirtoni.* We interpret these differences as a graviportal adaptation (i.e. adapted for weight bearing and slow locomotion). Furthermore, the manus appear to be more specialized for quadrupedal locomotion than in earlier diprotodontids and *Z. trilobus*, with greatly reduced grasping and food manipulation ability. The late Miocene to Pliocene saw the climate of Australia becoming cooler and drier, which drove the expansion of C_4_ grasslands and grassland-adapted herbivores [3,79,106–108]. Much of the Tirari Formation has been hypothesized to have been deposited during a time of increased aridity, and the composition of the Kanunka, Palankarinna and Toolapinna Local Faunas is mostly suggestive of at least a seasonally arid environment [20]. This may have resulted in diprotodontids, particularly browsers such as *Am. keanei*, needing to travel further for food and developing more efficient means of travelling between locations. The more open grassland habitats may have facilitated the specialization of limbs for increased vagility. Some adaptations in the limbs may also reflect increased body size among diprotodontids. However, size estimates based on humeral circumference for *Am. keanei* are smaller than those estimated for Alcoota diprotodontids (table 4), which may indicate that much of the morphology shared between *Am. keanei* and *D. optatum* are adaptations to walking as opposed to just weight bearing. *Diprotodon optatum* has been hypothesized to have been migratory [109] and perhaps *Am. keanei* was also. We were unable to infer the distribution of weight between the forelimbs and hindlimbs of *Am. keanei* because this requires the narrowest part of the diaphysis of most limb bones [38], and these were missing. This could be tested between more complete specimens of *D. optatum* (which has a similar adaptation to *Am. keanei*) and *Z. trilobus*, the latter displaying a morphology indicating its forelimb was adapted to a different function.

**Table 4.** Body size estimates using minimum humeral circumference of various diprotodontids taken from Richards *et al*. [38] with *Ambulator keanei* and Alcoota humeri inserted.

taxon	specimen	humeral circumference (mm)	mass estimate (kg)	upper estimate (kg)	lower estimate (kg)
*Ambulator keanei*	SAMA P54742	119	262.0	334.1	190.0
*Neohelos stirtoni*	QVM 1000GFV445	101	168.3	214.6	120.2
Alcoota diprotodontid	NTM P4114	166	632.1	806.0	458.3
Alcoota diprotodontid	SAMA P31863	93	137.4	175.2	99.6
Alcoota diprotodontid	NTM P5279	93	134.1	171.0	97.2
Alcoota diprotodontid	NTM P6449	110	211.9	270.2	153.7
Alcoota diprotodontid	NTM P4112	152	503.7	642.3	365.2
Alcoota diprotodontid	NTM P6764	184	835.5	1065.3	605.7
Alcoota diprotodontid	NTM P7251	1901	916.8	1169.0	664.7
*Diprotodon optatum*	SAM P5157	264	2187.0	2788.4	1561.5
*Diprotodon optatum* (mean data from Wroe *et al.* [1])	mean *n* = 9	249	1865.0	2377.9	1331.6
*Zygomaturus trilobus*	QVM 1992GFV246	160	577.2	735.9	412.1
*Zygomaturus trilobus*	TMAG Z3554	174	713.3	909.5	509.3

## 5. Conclusion

We describe the first postcranial material known for *Ambulator keanei*. This is only the fourth species of post-Miocene diprotodontid for which postcranial material has been described and includes the first three-dimensional description of the footpad morphology for the family. We found that *Am. keanei* expresses the earliest evidence for specialized graviportal locomotion within diprotodontids, suggesting that it may have been relatively vagile, which may correlate with late Pliocene grassland expansion. We also found evidence that the phalanges of graviportal diprotodontids were not used in weight bearing during locomotion. Systematic comparisons of established diprotodontid taxa indicate that inclusion of postcranial morphology in phylogenetic analyses is needed in order to resolve both species- and the higher-level taxonomy for the Diprotodontidae. Rare, well-preserved skeletal and soft tissue material found in the Main Body of the Tirari Formation indicates considerable potential for discovery of additional material. Future efforts should target collecting more vertebrate material from this formation. This may lead to not only a better understanding of marsupial diversity but a better understanding of the faunal change of Australia through time.

## Data Availability

All data including specimens used for morphological comparisons have been included in the paper or have been uploaded in the electronic supplementary material. Three-dimensional data of SAMA P54742 are available on Morphosource.org (Project ID: 000497863). The data are provided in the electronic supplementary material [110].
